# Electrostatic Regulation of Na^+^ Coordination Chemistry for High-Performance All-Solid-State Sodium Batteries

**DOI:** 10.1007/s40820-025-01910-1

**Published:** 2025-09-22

**Authors:** Penghui Song, Suli Chen, Junhong Guo, Junchen Wu, Qiongqiong Lu, Haijiao Xie, Qingsong Wang, Tianxi Liu

**Affiliations:** 1 The Key Laboratory of Synthetic and Biological Colloids, Ministry of Education, School of Chemical and Material Engineering, Jiangnan University, Wuxi, 214122 People’s Republic of China; 2 Institute of Materials, Henan Key Laboratory of Advanced Conductor Materials, Henan Academy of Sciences, Zhengzhou, 450046 People’s Republic of China; 3 Hangzhou Yanqu Information Technology Co., Ltd., Hangzhou, 310003 People’s Republic of China; 4 Bavarian Center for Battery Technology (BayBatt), Department of Chemistry, University of Bayreuth, Universitätsstr. 30, 95447 Bayreuth, Germany

**Keywords:** All-solid-state sodium metal batteries, Polymer electrolyte, Interfacial chemistry, Na^+^ transport kinetics, Electrostatic engineering

## Abstract

Ion migration capability and interfacial chemistry of solid polymer electrolytes (SPEs) in all-solid-state sodium metal batteries (ASSMBs) are closely related to the Na^+^ coordination environment. Herein, an electrostatic engineering strategy is proposed to regulate the Na^+^ coordinated structure by employing a fluorinated metal–organic framework as an electron-rich model. Theoretical and experimental results revealed that the abundant electron-rich F sites can accelerate the disassociation of Na-salt through electrostatic attraction to release free Na^+^, while forcing anions into a Na^+^ coordination structure though electrostatic repulsion to weaken the Na^+^ coordination with polymer, thus promoting rapid Na^+^ transport. The optimized anion-rich weak solvation structure fosters a stable inorganic-dominated solid–electrolyte interphase, significantly enhancing the interfacial stability toward Na anode. Consequently, the Na/Na symmetric cell delivered stable Na plating/stripping over 2500 h at 0.1 mA cm^−2^. Impressively, the assembled ASSMBs demonstrated stable performance of over 2000 cycles even under high rate of 2  C with capacity retention nearly 100%, surpassing most reported ASSMBs using various solid-state electrolytes. This work provides a new avenue for regulating the Na^+^ coordination structure of SPEs by exploration of electrostatic effect engineering to achieve high-performance all-solid-state alkali metal batteries.

**Graphical Abstract:**

**Supplementary Information:**

The online version contains supplementary material available at 10.1007/s40820-025-01910-1.

## Highlights

- An electrostatic engineering strategy is proposed to regulate the Na^+^ coordinated structure by employing a fluorinated metal–organic framework as an electron-rich model.
- The abundant electron-rich F sites can accelerate Na-salt disassociation while forcing anions into Na^+^ coordination structure though electrostatic effect to weaken the Na–O coordination, thus promoting rapid Na^+^ transport.
- Anion-rich weak Na^+^ solvation structure is achieved and contributes to a highly stable inorganic-rich solid–electrolyte interphase, significantly enhances the interfacial stability toward Na anode.
- Impressively, Na/Na symmetric cell delivered stable Na plating/stripping over 2500 h, and the assembled all-solid-state sodium metal batteries demonstrated stable performance of over 2000 cycles under high rate of 2 C with capacity retention nearly 100%.

**Supplementary Information:**

The online version contains supplementary material available at 10.1007/s40820-025-01910-1.

## Graphical abstract

**Figure:**
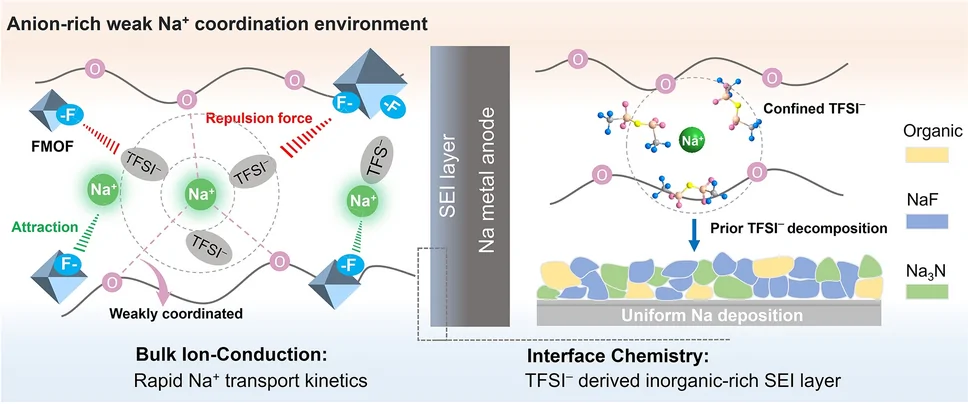

## Introduction

Developing all-solid-state sodium metal batteries (ASSMBs) is a vital way to eliminate potential safety risks and overcome the energy–density limitation faced by conventional liquid batteries [1–4]. Generally, solid-state electrolytes for all-solid-state batteries can be classified into inorganic solid electrolytes (ISEs) and solid polymer electrolytes (SPEs) [5, 6]. ISEs, such as oxide electrolytes, sulfide electrolyte, Na-β"-Al_2_O_3,_ etc., typically have high ionic conductivity, low electronic conductivity, and good mechanical properties, but their compatibility and interfacial contact with electrodes still need improvement [7–9]. In contrast, SPEs possess advantages of high flexibility, easy processability, and favorable interfacial contact with electrode, are considered to be ideal candidate materials for ASSMBs [10–12]. However, current SPEs suffer from low ionic conductivity and inferior interfacial compatibility with sodium metal anode, resulting in limited practical applications [13, 14]. As a commonly accepted cognition, the effective migration of ion in SPEs mainly occurs in the amorphous region of the polymer matrix, and a segmental motion of polymer chain promotes the Li^+^/Na^+^ hopping [15–17]. Therefore, current numerous research efforts in SPEs field have focused predominantly on increasing amorphous region to enhance ionic conductivity. For example, introducing inorganic fillers or organic plasticizers into SPEs to construct composite polymer electrolytes (CPEs) has been recognized as an effective strategy to decrease the crystallization region and facilitate the rapid ion transport. Despite some advances, the ionic conductivity of SPEs still cannot meet the actual commands, and less addressed problem of high interfacial resistance caused by unstable solid–electrolyte interphase (SEI) [18, 19].

Similar to the commonly accepted cognition in liquid electrolyte, the electrochemical properties of SPEs, including ion conduction behavior and interfacial chemistry on alkali metal anodes, are closely related to the coordination environment of alkali metal ions and ion–ion/ion–molecule interactions inside polymer electrolytes [20–22]. Taking the poly (ethylene oxide) (PEO)-based electrolyte as an example, in the typical solvation structure of PEO-based electrolyte, alkali metal ions dissociated from the counter ions and migrated through complexation and decomplexation with ether oxygen (EO) groups in PEO chains [23–25]. In this case, cations are strongly coordinated by polymer chains, resulting in the high desolvation energy barriers and sluggish ion migration kinetics [26, 27]. Moreover, this strong Na^+^-O coordination will lead to the complete solvation of Na^+^ by polymer, where the anions are expelled from the solvated structure, resulting in easy and prior decomposition of polymer matrix upon metallic anode to form an organic-rich SEI with poor electrochemical stability[28, 29]. Therefore, precise regulation of Na^+^ coordination environment in the SPEs will be an effective strategy to improve ion conduction capability and achieve stable sodium anode/electrolyte interface with low resistance.

**Fig. 1:**
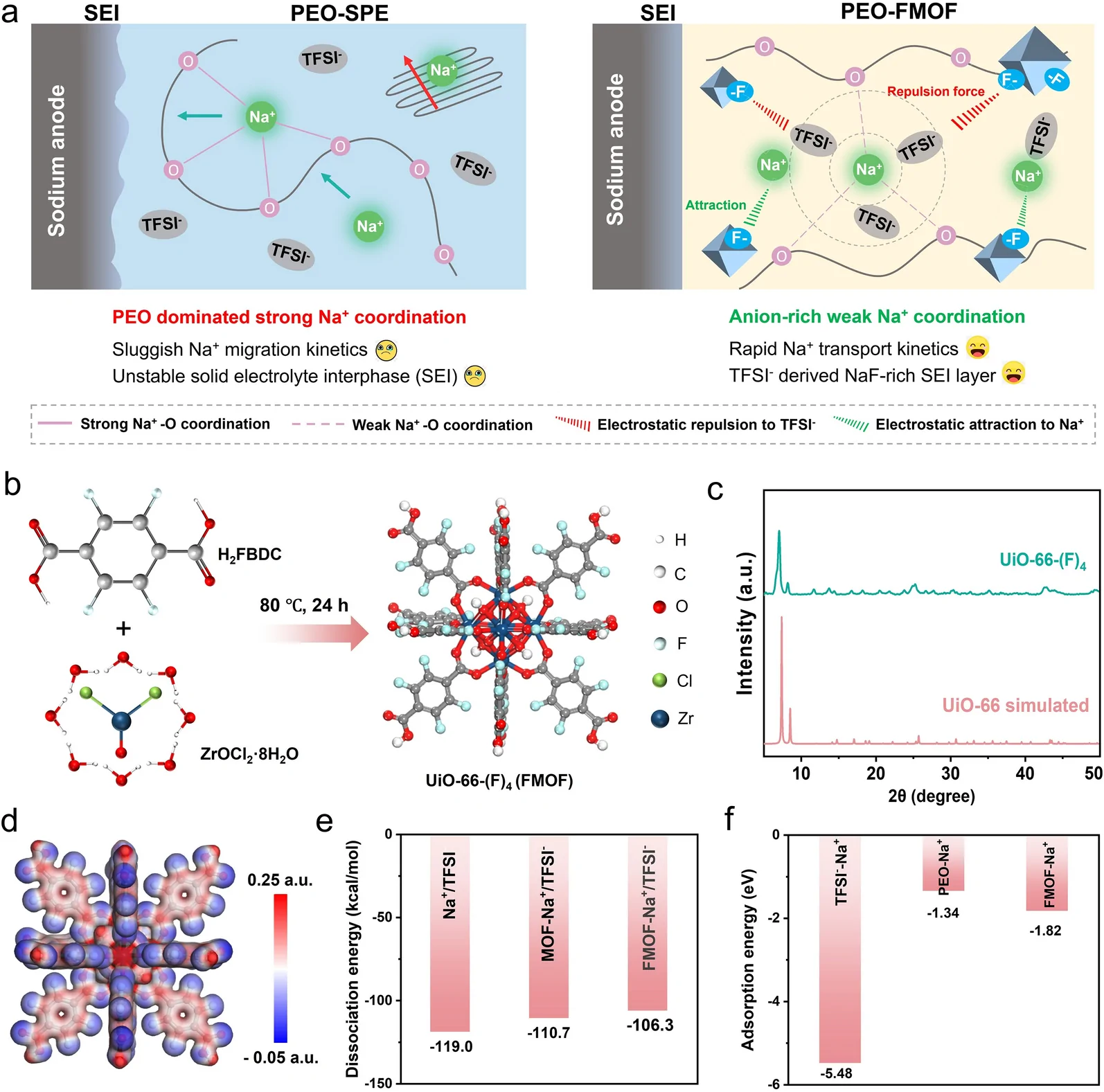
Schematic illustration of Na^+^ coordinated structure modulation and characterization of FMOF. **a** Scheme diagrams of Na^+^ coordination in PEO-SPE and PEO-FMOF. **b** Diagram of synthetic process for FMOF. **c** XRD spectra of the as-prepared FMOF. **d** Optimized geometry and electrostatic potential of FMOF. **e** Calculated dissociation energy of Na^+^ to TFSI − under three different conditions. **f** Adsorption energies of Na^+^ in three different environments

In this study, a fluorinated metal–organic framework UiO-66-(F)_4_ (FMOF) acted as an electron-rich model was synthesized and introduced into PEO-base electrolyte to modulate the Na^+^ coordination environment. The abundant fluorine atoms exposed on the FMOF surface make it a powerful electron-rich centrosome. Based on the electrostatic attraction with Na^+^ ions and repulsion with TFSI^−^ anions, FMOF added in PEO-based electrolyte facilitates the dissociation of sodium salts, while forcing the anions to participate in the solvation structure of Na^+^ (Fig. 1a). This leads to the formation of an anion-rich Na^+^ solvated structure, significantly weakening the Na^+^ coordination with PEO chains, thus promoting the rapid Na^+^ migration, showing a significantly increased ionic conductivity of up to 1.01 mS cm^−1^ and Na^+^ transference number of 0.78. In addition, the confined TFSI^−^ anions in solvated structure readily decompose on the anode surface, this eventually leads to a stable inorganic-rich SEI for uniform Na deposition. Accordingly, the symmetric Na/Na cells employed this FMOF-modified PEO-based electrolyte (PEO-FMOF) could stably operate more than 2500 h under 0.1 mA cm^−2^, while the ASSMBs show an outstanding cycling performance, delivering a long lifespan for over 2000 cycles with capacity retention up to 100% at 2 C. Our work provides vital insight into the electrostatic engineering in effective modulation of Na^+^ coordination environment of SPEs in developing high-performance ASSMBs.

## Experimental Section

### Materials

Zirconyl chloride octahydrate (ZrOCl_2_·8H_2_O, 99.9%), tetrafluoro terephthalic acid (H_2_fBDC, 97.0%), poly (ethylene oxide) (PEO, average Mw = 600,000 g mol^−1^), and anhydrous acetonitrile (99.8%) were obtained from Aladdin Chemical Reagent Co. Ltd., China. Acetic acid (99.5%), NaTFSI, and molecular sieves type 4 A (Ф3–5 mm) were purchased from Sinopharm Chemical Reagent Co. Ltd. Methanol (99.5%) was dried with 4 A molecular sieves before use.

### Synthesis of UiO-66-(F)_4_

The synthesis of UiO-66-(F)_4_ was partially modified and adjusted based on reported work. In the initial synthesis procedure, ZrOCl_2_·8H_2_O (5.2 mmol) and H_2_fBDC (5 mmol) were suspended in 50 mL of water/acetate acid (3:2 in volume) mixed solvent for UiO-66-(F)_4_. The solution was stirred at room temperature for 1 h and then heated at 80 °C under reflux for 24 h. The white suspended solid UiO-66-(F)_4_ was collected by centrifugation and washed with methanol for 3 days using Soxhlet extraction method, during which time the solvent was decanted, and fresh methanol was replaced every 24 h. Finally, the product was treated through freeze-drying technique and followed by further drying at 120 °C under vacuum for 24 h.

### Preparation of PEO-FMOF and PEO-SPE Membranes

Our designed fluorinated solid composite polymer electrolyte was prepared by adding fluorinated MOFs into PEO-based polymer electrolyte. Firstly, the PEO polymer and NaTFSI were dissolved into 20 mL anhydrous acetonitrile to form a transparent homogenous solution, where the EO/Na^+^ ratio was controlled at 16:1. Then, an appropriate amount of UiO-66-(F)_4_ was added to the solution and stirred to form a uniform suspension. After that, the suspension was casted on a PTFE plate and dried overnight in a vacuum oven at 60 °C. In addition, electrolyte membranes with different MOF mass concentrations were prepared to select the optimal concentration. Pure PEO-based polymer electrolyte was also prepared by using conventional solution-casting method to act as control samples. All preparation processes of the SPEs were operated in an Ar-filled glove box (H_2_O < 0.01 ppm and O_2_ < 0.01 ppm).

### Materials Characterization

Fourier transform infrared (FT-IR, Nicolet 6700) spectra were recorded using the attenuated total reflectance (ATR) technique from 4000 to 650 cm^−1^. Field emission scanning electron microscope (SEM, Hitachi S-4800) with energy-dispersive spectrometry (EDS) was used to acquire the microstructure and morphologies of UiO-66-(F)_4_ and electrolyte membranes (sputtered with Au for 30 s). The crystal structure of samples was revealed by X-ray diffraction (XRD, Bruker D8) measurement which was operated at 40 mA and 40 kV with a copper target (λ = 1.54 Å, the scanning rate was 10° min^−1^). The specific surface area was calculated based on the Brunauer–Emmett–Teller (BET, Quantachrome Autosorb NOVA 2200e) with N_2_ adsorption–desorption measurements. The Raman spectroscopy (inVia Micro Confocal) tests were carried out from 720 to 780 cm^−1^. The thermal stability of sample membranes was studied via thermogravimetric analysis on the thermal gravimetric analysis (TGA, TGA2) instrument under N_2_ flow at a ramp rate of 10 °C min^−1^ from 30 to 800 °C. The melting temperatures of electrolyte membranes were analyzed by different scanning calorimetry (DSC, DSC3) from −70 to 80 °C at a ramp rate of 10 °C min^−1^. Atomic Force Microscope (AFM, Multimode 8) was employed to measure the topography and Young’s modulus of the electrolyte. A tensile testing machine was used to obtain the stress–strain curve of the PEO-SPE and PEO-FMOF. The elemental composition of electrolyte membranes was analyzed by X-ray photoelectron spectrometer (XPS, Kratos Analytical Inc.). ^23^Na solid-state NMR spectra were detected on a Bruker Avance Neo 400WB with a 3.2 mm DVT probe.

### Electrochemical Measurements

The ionic conductivity (*σ*) of the electrolyte was estimated by electrochemical impedance spectroscopy (EIS) with a frequency range from 0.01 to 10^6^ Hz, using a polished stainless-steel as a blocking electrode. The ionic conductivity was calculated according to the following equation:

1 $$\sigma =\frac{L}{\mathrm{SR}}$$

where *L* (cm) represents the thickness of the electrolyte, *R* (Ω) is the bulk resistance of the solid–electrolyte membrane derived by EIS, and S (cm^2^) refers to the area of the stainless-steel electrode.

The activation energy (*E*_*a*_) of Na^+^ migration in electrolyte membranes was calculated according to the following Vogel–Tamman–Fulcher (VFT) empirical equation:

2 $${\sigma _T} = \frac{A}{{{T^{{\raise0.7ex\hbox{$1$} \!\mathord{\left/
 {\vphantom {1 2}}\right.\kern-\nulldelimiterspace}
\!\lower0.7ex\hbox{$2$}}}}}}{\rm{exp}}\left( { - \frac{{{E_a}}}{{T - {T_0}}}} \right)$$

where *σ*_*T*_ is the ionic conductivity (S cm^−1^), T is the absolute temperature (K), *E*_*a*_ is the activation energy (eV) of activated ion-hopping conduction process, A is the pre-exponential factor, which consists of three parts: the carrier concentration, the lepton distance, and the ionic lepton frequency, and *T*_0_ is the ideal glass transition temperature, which takes the value of Tg-50 K, and *T*_*g*_ is the glass transition temperature measured by DSC.

The electrochemical stability window was evaluated with Na/SS cells by linear sweep voltammetry (LSV), which was conducted on a CHI660E electrochemical station in the voltage range of 2–7 V at a potential scanning speed of 0.5 mV s^−1^.

The Na^+^ transference number (${t}_{{\mathrm{Na}}^{+}}$) of electrolytes was examined in symmetric Na/Na cells by an Autolab electrochemical working station. The symmetric cells were polarized with an AC voltage of 10 mV, and the EIS spectra before and after polarization were also measured. The ${t}_{{\mathrm{Na}}^{+}}$ was calculated based on the following formula:

3 $${t}_{{\mathrm{Na}}^{+}}=\frac{{I}_{S}(\Delta V-{I}_{0}{R}_{0})}{{I}_{0}(\Delta V-{I}_{S}{R}_{S})}$$

where *I*_S_ and *I*_0_ separately represent the initial and steady current through the cell, and *R*_0_ and *R*_*S*_ are the interfacial resistance before and after polarization, respectively. ∆*V* is the applied constant potential.

The NaV_2_(PO_4_)_3_ (NVP) cathode was prepared by mixing NVP, Super P, and PVDF in N-methyl-2-pyrrolidinone with a weight ratio of 8:1:1. The slurry was coated on Al foil and dried at 120 °C for 12 h under vacuum. Here, the specific density of NVP active material is approximately 1.16 mg cm^−2^. The all-solid-state NVP/Na batteries were assembled without the addition of any liquid electrolyte. The galvanostatic charge/discharge tests were carried out at Land CT2001A battery instrument between 2.5 and 3.8 V under different current densities (1 C = 120 mAh g^−1^). The sample preparation and cell assembly were performed in an argon-filled glove box.

## Results and Discussion

### Synthesis and Characterization of FMOF

The fluorinated MOF, UiO-66-(F)_4_ (FMOF), was synthesized according to a previously reported work with some modification [30], the preparation process and chemical structure of FMOF is shown in Fig. 1b. The crystal structure of as-synthesized FMOF was confirmed by XRD, the characteristic peaks of FMOF fit well with the simulated UiO-66 (Fig. 1c). The XPS spectra in Fig. S1 confirm the presence of Zr and F in FMOF, further indicating the successful preparation of FMOF. Figure S2 presents the morphologies of FMOF that were examined using SEM. The FMOF has a cubic nanostructure with the particle size of around 300 nm, which helps the fillers to well composite with the polymer matrix. Additionally, as seen from the N_2_ adsorption–desorption isotherm test in Fig. S3, the FMOF shows a high specific surface area of 417.7 m^2^ g^−1^ with pore diameters less than 10 nm, implying enough interaction area with sodium salt in polymer matrix (PEO-NaTFSI).

As shown from Fig. 1d, the optimized geometry and electrostatic potential (ESP) reveal that the introduction of fluorine atoms engenders negative charge accumulation at respective polar sites (blue colored region), which will promote sodium salt dissociation and present electrostatic repulsion for anion [31]. Further, taking FMOF as a proof of electron-rich centrosome concept, we performed density function theory (DFT) calculations to probe the effect of FMOF on the dissociation of Na salts and the Na^+^ adsorption. In order to exclude the effect of Lewis-acidic Zr metal sites in MOF on the dissociation behavior of sodium salt, the typical UiO-66 without fluorine atoms was also provided for comparison. It was found that the dissociation energies between TFSI^−^ and Na^+^ in three different environments are calculated to be −106.3 kcal mol^−1^ for UiO-66-(F)_4_, −110.7 kcal mol^−1^ for UiO-66 and −119.0 kcal mol^−1^ for initial NaTFSI salt (Fig. 1e). Moreover, the adsorption energy of FMOF-Na^+^ (−1.82 eV) is higher than that of EO groups in PEO chain (−1.34 eV), lower compared to TFSI^−^ (−5.48 eV) (Fig. 1f). These results suggest that the introduction of FMOF presents the potential for assisting the salt dissociation, thus contributing to high concentration of free Na^+^ in electrolytes.

### Physicochemical Properties and Electrochemical Performance of PEO-FMOF

**Fig. 2:**
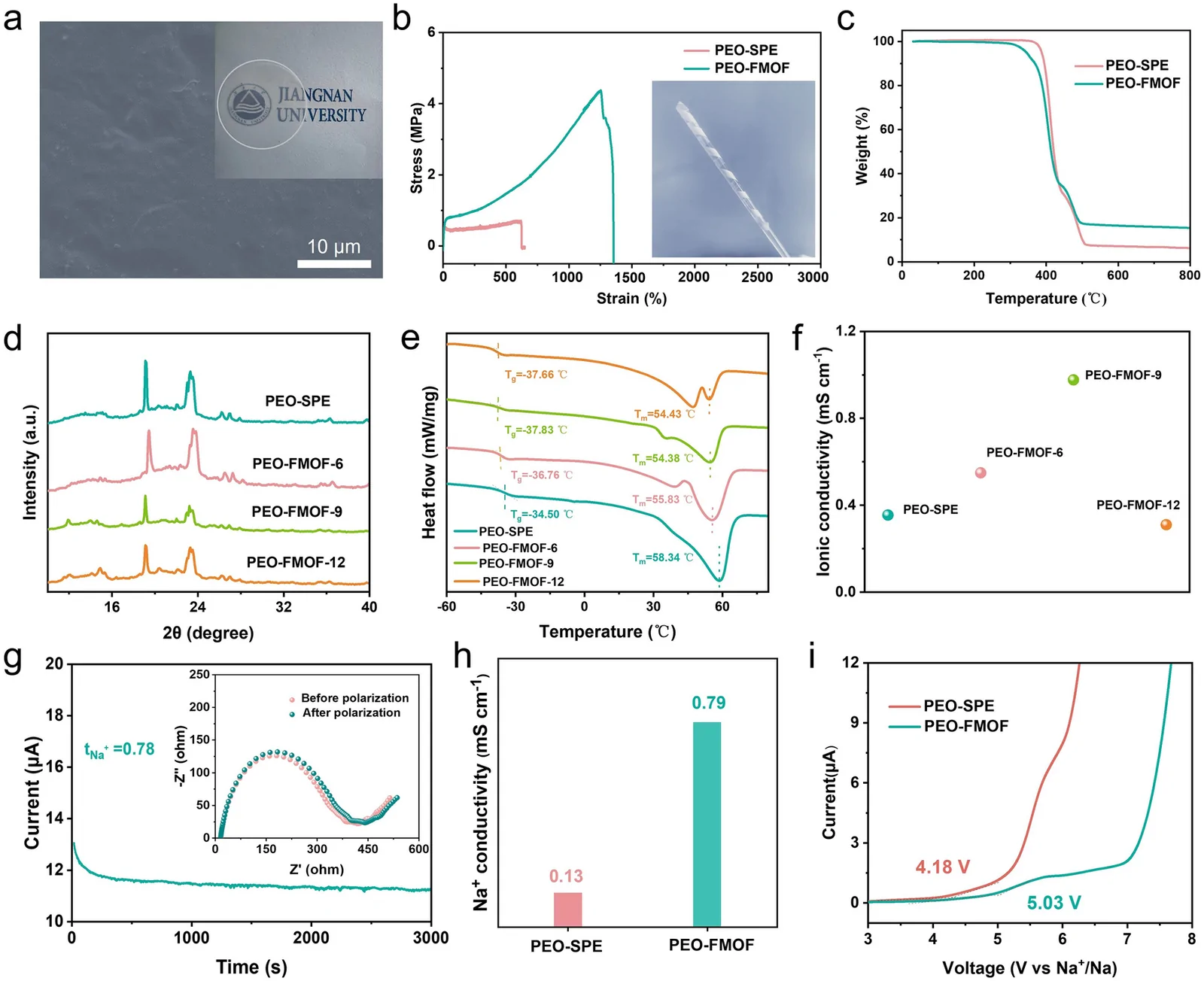
Physical properties and electrochemical performances of PEO-FMOF. **a** SEM of PEO-FMOF (inset is digital picture of PEO-FMOF). **b** Stress–strain curves of PEO-SPE and PEO-FMOF. **c** TGA curves of the PEO-SPE and PEO-FMOF. **d** XRD patterns, **e** DSC profiles, and **f** ionic conductivity at 60 °C of PEO-SPE and PEO-FMOF with different FMOF mass concentrations. **g** Polarization curve of PEO-FMOF (inset is fitted EIS before and after polarization of cell). **h** Na^+^ conductivity of PEO-SPE and PEO-FMOF. **i** LSV curves of PEO-SPE and PEO-FMOF

The PEO-FMOF membrane was obtained by a typical solution-casting technique. As observed from Fig. 2a, the PEO-FMOF unveils a smooth surface morphology without apparent particle aggregation in contrast with PEO-SPE featuring a rough surface (Fig. S4). Meanwhile, from the inset of Fig. 2a, the PEO-FMOF still presents translucent appearance after introduction of FMOF, indicating excellent chemical compatibility of FMOF in polymer matrix. In Fig. S5, EDS mapping of PEO-FMOF shows that F, Zr, and Na elements are uniformly distributed on the SPE, further demonstrates the high compatibility. Figure 2b indicated that the PEO-FMOF exhibits excellent mechanical properties, yields a tensile strength of 4.36 MPa and elongation at break of 1350%. In addition, PEO-FMOF also shows excellent tensile properties, which can be easily stretched without breaking (Fig. S6). From the AFM data in Figs. S7 and S8, the surface of PEO-FMOF becomes smoother, ensuring a favorable electrolyte and electrode contact. Meanwhile, Young’s modulus of PEO-FMOF is much higher than PEO-SPE, which is beneficial for suppressing the growth of Na dendrites [32]. The considerable mechanical behavior of the PEO-FMOF may arise from the intermolecular interactions between the nanofillers and the PEO matrix. This not only prevents the agglomeration of the FMOF nanofillers to achieve stress dispersion, but also forms a physically cross-linked network structure, which further enhances the tensile strength [33]. In addition, TGA indicated that the PEO-FMOF exhibited good thermal stability with thermal degradation temperature up to 300 °C, guaranteeing the wide-temperature range safety of the resulting ASSMBs.

Next, the PEO-FMOF with different FMOF contents was prepared to screen the optimal mass concentration and marked as PEO-FMOF-x (x represents the mass concentration of FMOF). XRD analysis in Fig. 2d showed reduced intensity of characteristic peaks for PEO in FMOF-6, FMOF-9, and FMOF-12, compared to that in PEO-SPE. The dramatically reduced crystal enthalpy of PEO-FMOF electrolytes indicates the decreased crystallization, proving the high plasticizing effect of FMOF for increasing the amorphous region of PEO, thus facilitating the rapid Na^+^ transport [34]. DSC was further performed to measure the glass transition temperature (*T*_*g*_) and the degree of crystallization. As depicted in Fig. 2e, compared to PEO-SPE, the PEO-FMOF samples deliver both lower temperature of *T*_*g*_ and melting points (*T*_*m*_), implying the facilitated free mobility of PEO chain. Furthermore, the crystallinity of each sample using the complete melting enthalpy of standard PEO was calculated as provided in Table S1. It was found that PEO-FMOF-9 had the lowest crystallinity, indicating that the addition of FMOF increased the amorphous region of PEO. The ionic conductivity of different electrolytes was measured and is shown in Fig. S9, the introduction of FMOF facilitates the ionic migration of PEO-SPE. When the FMOF content is up to 9 wt%, the PEO-FMOF electrolyte displays the highest ionic conductivity of 1.01 mS cm^−1^ at 60 °C (Fig. 2f), which is much higher than that of PEO-SPE (0.35 mS cm^−1^). Excessive FMOF may cause agglomeration and reduce the free volume of the polymer [35], thus decreasing the ionic conductivity when the FMOF content is over 9 wt%. Meanwhile, as shown in Fig. S10, the composite electrolyte prepared using 9 wt% FMOF has a lower activation energy compared to PEO-SPE (0.71 vs. 1.02 eV). Apart from the ionic conductivity, ${t}_{{\mathrm{Na}}^{+}}$ is another key factor for SPE [36]. It was found that the PEO-FMOF electrolyte exhibits a much higher ${t}_{{\mathrm{Na}}^{+}}$ of 0.78 relative to the PEO-SPE counterpart (0.36) (Figs. 2g and S11). As indicated in Fig. 2h, owing to the high ionic conductivity and ${t}_{\mathrm{Na}^{+}}$, the Na^+^ conductivity of the PEO-FMOF is up to 0.79 mS cm^−1^, implying the key role of the FMOF in enhancing Na^+^ migration. The electrochemical stability window of the electrolytes was estimated by LSV test (Fig. 2i). The PEO-FMOF shows a decomposition potential up to 5.03 V vs. Na^+^/Na, suggesting its broadened electrochemical stability for practical application in high-voltage batteries. This wider electrochemical window may be attributed to the physical barrier effect of FMOF itself and the reduction of electron cloud density on polymer segments through Lewis acid–base interactions between abundant metal sites of FMOF and oxygen atoms of PEO chains, making the polymer more difficult to oxidize.

### Exploring the Transport Behavior of Na^+^ in the PEO-FMOF

**Fig. 3:**
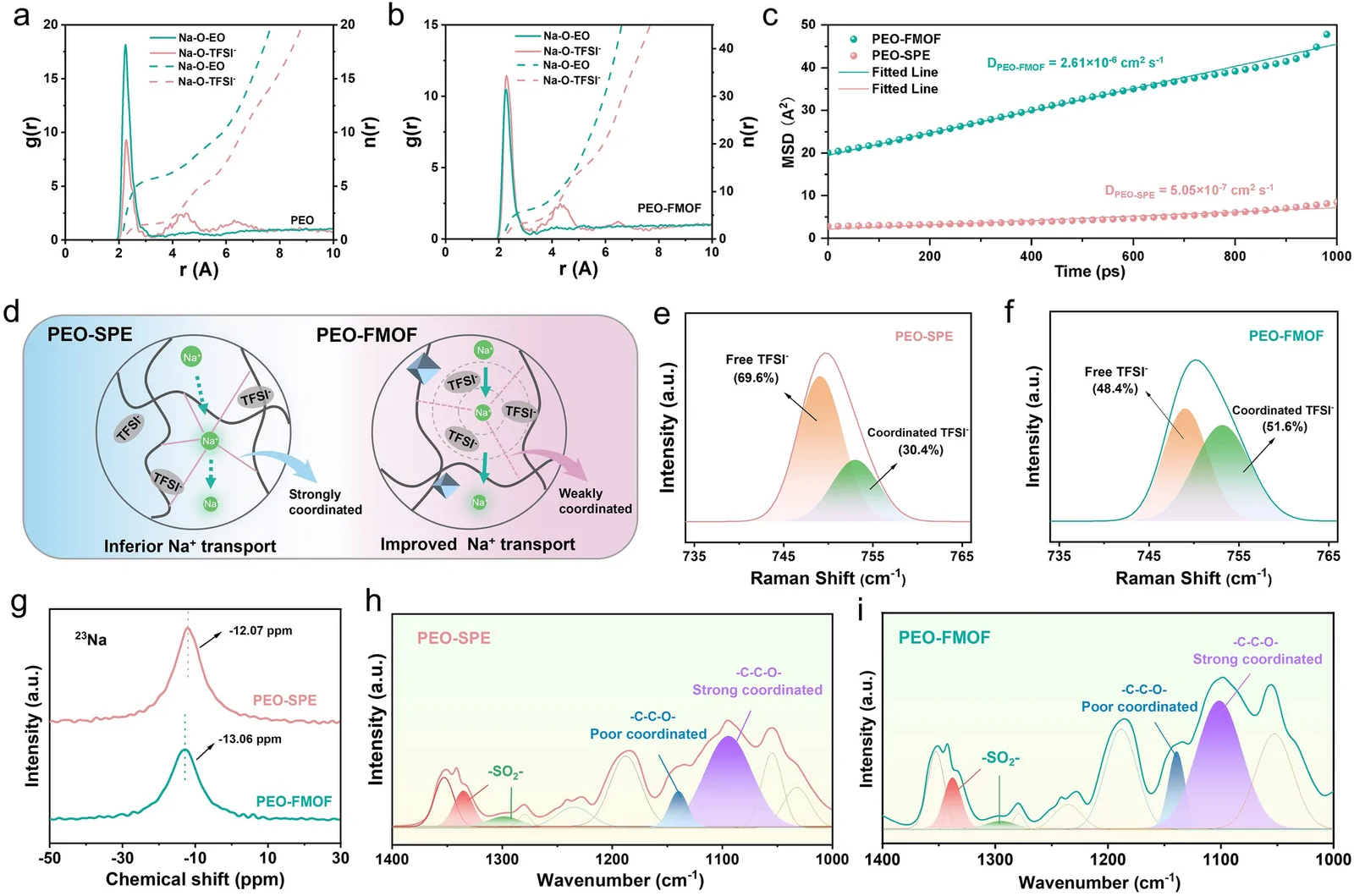
Exploring the mobility of sodium ions in the PEO-FMOF. **a**, **b** Na–O radial distribution functions (RDF) of PEO-SPE and PEO-FMOF. **c** Mean square displacement of Na^+^ in the PEO-SPE and PEO-FMOF. **d** Schematic illustration of the detailed coordination structure of Na^+^ in the PEO-SPE and PEO-FMOF. **e**, **f** Raman spectra of the PEO-SPE and PEO-FMOF. **g** Solid-state NMR of the electrolytes. **h**, **i** FT-IR spectra of PEO-SPE and PEO-FMOF

The radical distribution functions (g(r), solid lines) and coordination numbers (n(r), dash lines) of Na^+^-O were further calculated to confirm the detailed coordination structure of Na^+^ in different electrolytes (Fig. S12). As shown in Fig. 3a, b, PEO-SPE-Na displays stronger intensity of the dominated peak at 2.2 Å than PEO-FMOF-Na, indicating that the Na^+^-O (EO) bonds in PEO-FMOF-Na are weakened, and more bonded Na^+^ are released. The n(r) in PEO-FMOF-Na is also lower than the PEO-SPE-Na, which indicates a much weaker polymer-Na^+^ coordination environment. Notably, compared to PEO-SPE and PEO-MOF (composite electrolyte using fluorine-free MOF filler) systems (Fig. S13), the intensities of the dominated peaks for Na^+^-O (TFSI^−^) display an obvious increase in PEO-FMOF-Na, indicating that more anions enter into the Na^+^ coordinated sheath, resulting in the formation of an anion-rich weak Na^+^-O coordination environment. Based on this, a higher Na^+^ diffusion coefficient was obtained in the PEO-FMOF (Fig. 3c). This may be attributed to the electrostatic repulsive interaction between the FMOF and TFSI^−^. As Fig. 3d shown, in the PEO-FMOF system, the abundant TFSI^−^ anions are forced into the Na^+^ coordinated sheath to form an anion-rich coordinated structure, which will not only inevitably weaken the Na^+^-O coordination between Na^+^ and EO groups in PEO chains, but also facilitate the formation of inorganic component-rich SEI upon sodium metal anode [37].

Since the S–N–S bending in TFSI^−^ is sensitive to cation–anion complexation, the solvation structures of Na^+^ were further studied by Raman spectroscopy (Figs. 3e, f and S14). The signals around at 748 and 753 cm^−1^ are assigned to the free TFSI^−^ and coordinated TFSI^−^, respectively [38]. A much higher integral intensity was detected of the coordinated TFSI^−^ peak in PEO-FMOF compared to those of PEO-SPE and PEO-MOF samples. This manifests that more TFSI^−^ anions are confined in the coordinated sheath after introducing FMOF, weakening solvating ability of PEO surrounding Na^+^. ^23^Na solid-state nuclear magnetic resonance (ss-NMR) spectra were recorded to specify the distinct Na chemical environments in the PEO-SPE and PEO-FMOF systems (Fig. 3g). Clearly, sodium nuclei in PEO-SPE are most shielded with a positive shift (−12.07 ppm) because of the strong coordination between Na^+^ and adjacent ethers. In contrast, sodium nuclei in PEO-FMOF are less shielded with a chemical shift of −13.06 ppm, suggesting that the addition of FMOF destroys the strong binding between Na^+^ and PEO chains. This further corroborates superior Na^+^ mobility in the PEO-FMOF system. FT-IR was performed to further verify the positive modulation of FMOF on Na^+^ coordinated structure. As shown in Fig. 3h, i, the peaks at 1335 and 1299 cm^−1^ are assigned to the asymmetric -SO_2_- in PEO-SPE, while for the PEO-FMOF system, the peaks shift to 1338 and 1294 cm^−1^, suggesting the enhanced interaction between Na^+^ and O atoms in the sulfonic acid groups of TFSI^−^ anions [39]. Meanwhile, the characteristic peaks of C–O–C vibration at around 1050 ~ 1150 cm^−1^ can be divided into the coordinated C–O–C vibration and the free C–O–C vibration [40]. When introducing FMOF into polymer matrix, the coordinated C–O–C vibration is significantly reduced, demonstrating the weakened solvating ability of PEO chains to Na^+^ and more TFSI^−^ anions into the coordinated structure.

### Electrochemical Stability of the Na/Na Symmetric Cells

**Fig. 4:**
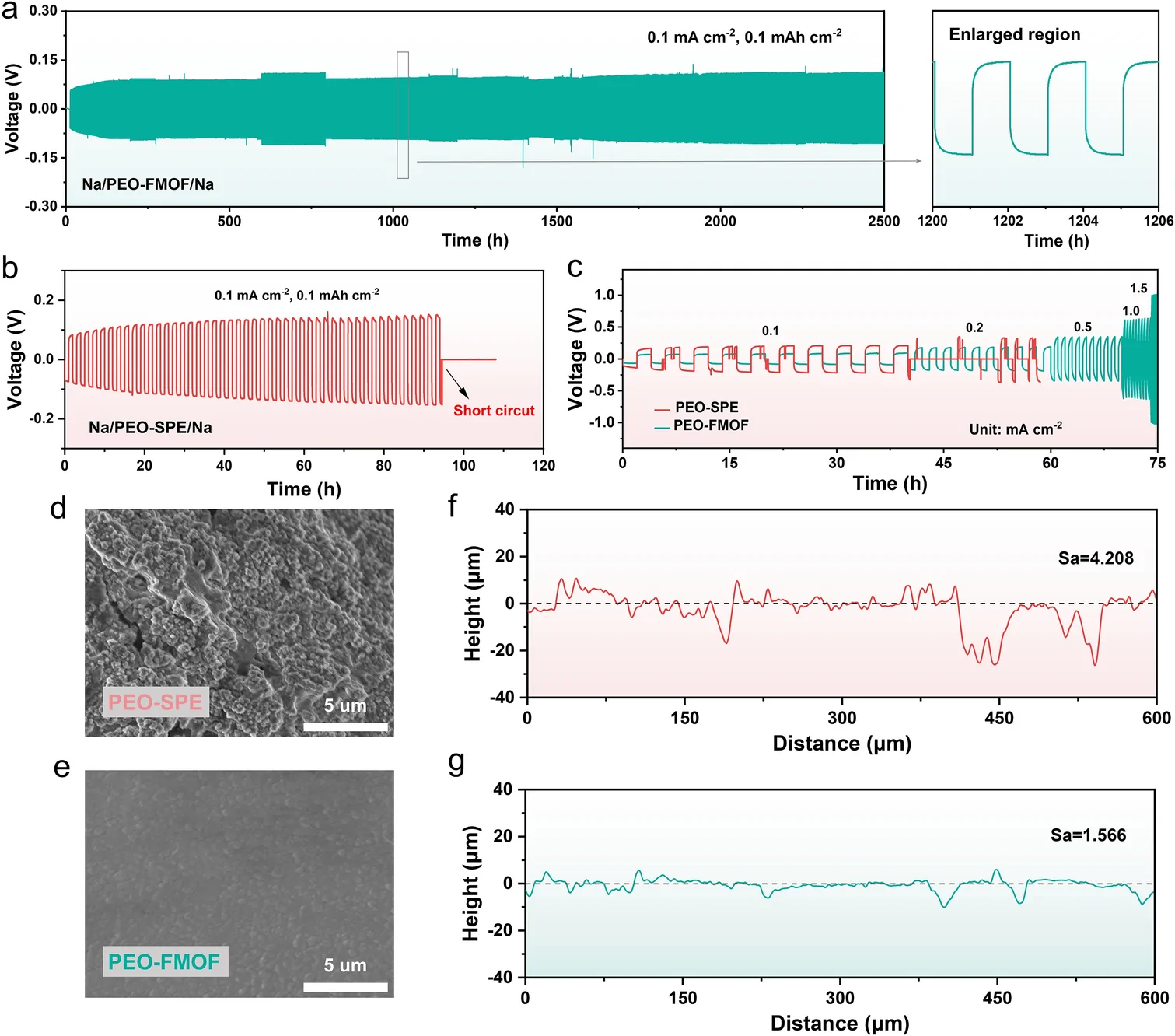
Electrochemical performance of Na symmetric cells and deposition behavior of Na anode. **a**, **b** Long-term cycling performance of Na/Na symmetric cells using PEO-FMOF and PEO-SPE at 0.1 mA cm^−2^ and 0.1 mAh cm^−2^. **c** Voltage profiles of repeated Na plating/stripping in different symmetric cells at different current densities. **d**, **e** SEM images of the cycled Na anode surfaces disassembled from symmetric cell using PEO-SPE and PEO-FMOF. **f**, **g** Three-dimensional CLSM height images of the PEO-SPE and PEO-FMOF dissembled from the Na/Na symmetric cells

To study the electrochemical properties of the polymer electrolytes with different Na^+^ solvation structures (PEO-SPE and PEO-FMOF), we constructed Na/Na symmetric cells for galvanostatic cycling tests. Figure 4a, b shows the long-term cycling performance of Na/Na symmetric cells using PEO-FMOF and PEO-SPE at 0.1 mA cm^−2^ and 0.1 mAh cm^−2^. It can be seen that the polarization voltage of the Na/PEO-SPE/Na cell is continuously increased, and finally has a short circuit after cycling for 90 h due to the inferior interfacial stability between PEO-SPE and sodium metal anode. In contrast, the cell with PEO-FMOF displays a long-cycling life of over 2500 h without short-circuit occurrence. A sudden increase in voltage at around 600 h and decrease at around 1400 h may be caused by the slight temperature fluctuations. In addition, the symmetric cell with PEO-FMOF also shows a long-cycling stability of more than 300 h at 1.5 mA cm^−2^ and 1.5 mAh cm^−2^ (Fig. S15). Due to the superiority of PEO-FMOF in enhancing the interfacial compatibility toward Na metal anode, a better rate capability was achieved for Na/PEO-FMOF/Na symmetric cell in the range of current density from 0.1 to 1.5 mA cm^−2^ (Fig. 4c).

In order to explain the phenomena of stable cycling performances and uniform sodium deposition, we investigated the surface morphology of cycled sodium anodes disassembled from different symmetric cells. As shown from Fig. 4d, the cycled sodium metal from PEO-SPE-based cell displays an irregular surface with uneven particles and aggregations were found, proving the failure of the Na/PEO-SPE/Na cell may be caused by the permeation of sodium dendrites. By contrast, the surface of the sodium anode with PEO-FMOF electrolyte is relatively smooth and flat (Fig. 4e), implying that the PEO-FMOF possesses an enhanced Na plating/stripping process and excellent dendrite suppression capability. In addition, the corresponding surface profile of cycled electrolyte membrane is further analyzed by 3D confocal laser scanning microscope (CLSM). Compared with PEO-SPE (Figs. 4f and S16a), PEO-FMOF has flatter surface fluctuation and lower surface roughness (*S*_*a*_) of 1.566 μm (Figs. 4g and S16b), also indirectly proves that a dense sodium deposition was achieved for PEO-FMOF-based sodium anode, which is conductive to improving the cycle life of cells.

**Fig. 5:**
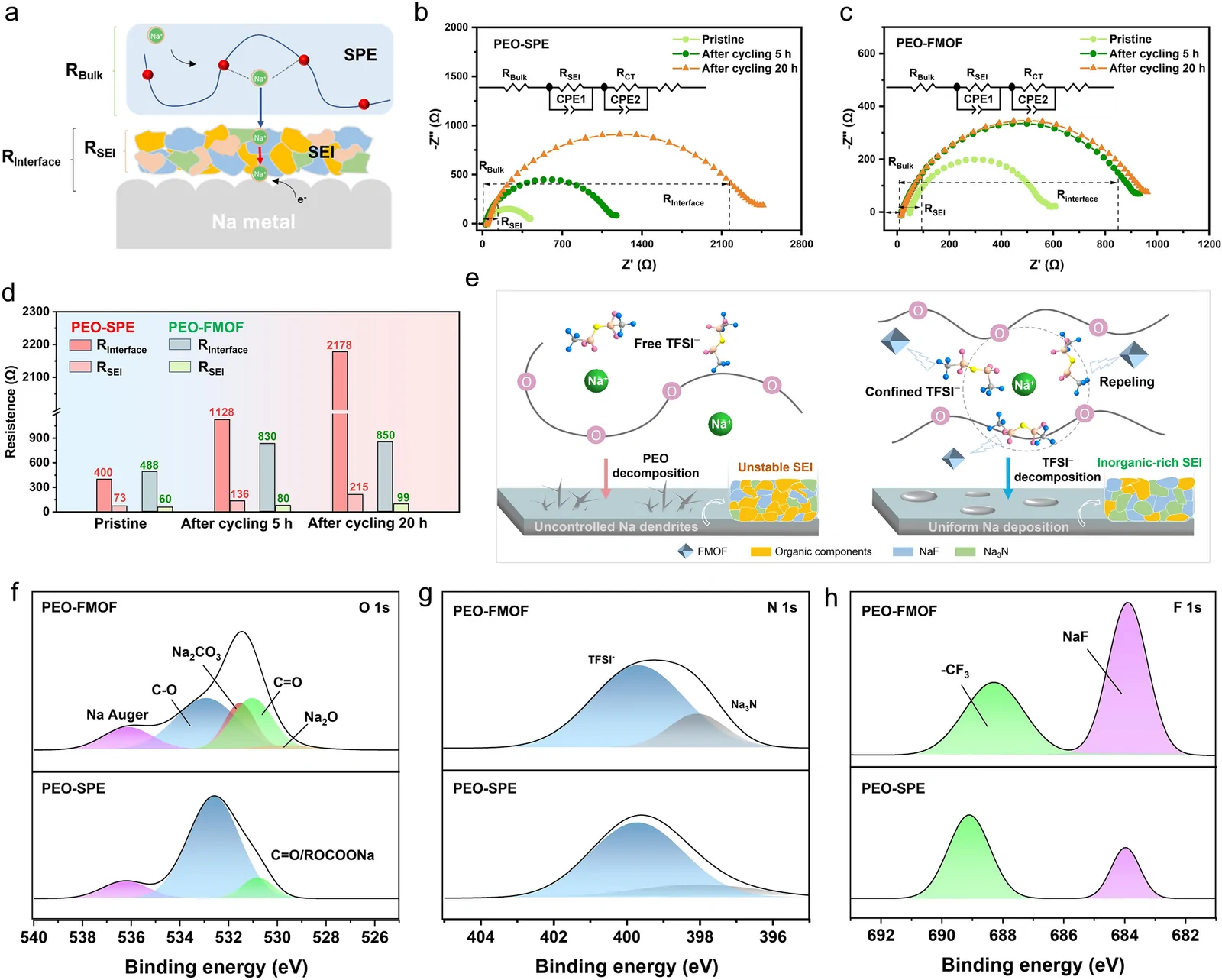
Interfacial chemistry between the PEO-FMOF and Na anodes. **a** Na^+^ deposition behavior at sodium metal surface and electrochemical resistance distribution during battery charge/discharge cycling. **b**, **c** Typical Nyquist plots of Na/PEO-SPE/Na and Na/PEO-FMOF/Na symmetric cells at different cycling times. **d** Interface resistance R_Interface_ and SEI resistance R_SEI_ comparison of PEO-SPE- and PEO-FMOF-based symmetric cells. **e** Schematic representation of SEI formation at molecular level. **f–h** XPS spectra of Na foils after cycling with the PEO-SPE and PEO-FMOF: O 1*s*, N 1*s*, and F 1*s*

As a consensus, the Na^+^ migration behavior on sodium metal surface can be divided into the following steps: (1) Na^+^ transport in polymer matrix; (2) Na^+^ desolvation in the SEI layer; and (3) Na^+^ passing through the SEI layer and deposited on the sodium metal surface, as shown in Fig. 5a [41]. In a symmetric Na/Na cell, the interfacial migration kinetics of Na^+^ can be studied by EIS, with the ultra-high-frequency region described as the polymer mass-transfer resistance (*R*_Bulk_). Na^+^ transport across the SEI (*R*_SEI_) and charge transfer impedance (*R*_CT_) during desolvation often overlap at mid-frequency and high-frequency regions to form the surface resistance (*R*_Interface_).

In order to better investigate the changes in electrochemical properties of SEI, we performed ex situ EIS measurements on symmetric cells at different cycling times at 0.1 mA cm^−2^ and 0.1 mAh cm^−2^. The Nyquist plot shows the impedance changes of Na/Na symmetric cells with PEO-SPE and PEO-FMOF before and after cycling (Fig. 5b, c). By comparing the impedance changes in the pristine and cycling after a period of time, the chemical stability of different electrolytes to sodium metal can be observed. Compared with initial state, the *R*_SEI_ and *R*_Interface_ of PEO-SPE and PEO-FMOF both increased after cycling (Fig. 5d), which may be due to the inevitable partial decomposition of polymer matrix at the interface. However, after cycling 20 h, the *R*_SEI_ and *R*_Interface_ of PEO-SPE-based symmetric cell significantly increased to 215 Ω and 2178 Ω, while the PEO-FMOF kept relatively stable, showing smaller *R*_SEI_ and *R*_Interface_ of only 99 Ω and 850 Ω, respectively. The explanation for this result could be the presence of more free TFSI^−^ anions within the PEO-SPE electrolyte, leading to their ejection from the PEO solvated structure, which makes the prior decomposition of the PEO upon sodium anode and generates an organic-rich SEI layer with inferior electrochemical stability [42]. In contrast, within the PEO-FMOF electrolyte, due to the formation of anion-rich coordination structure, more TFSI^−^ anions are incorporated into the solvation sheath, resulting in their preferential decomposition and the formation of an inorganic-rich SEI layer with superior electrochemical stability to induce uniform Na deposition, as depicted in Fig. 5e.

Next, XPS was further performed to analyze the chemical composition of SEI layer on the sodium anode surface after cycling with different electrolytes. In the C 1*s* spectra (Fig. S17) and O 1*s* spectra (Fig. 5f), characteristic peaks for C–O, C = O, and ROCOONa groups were observed in the PEO-SPE and PEO-FMOF. These organic components mainly originate from the oxidative decomposition of PEO in electrolytes [43], with low strength in PEO-FMOF and relatively high in PEO-SPE, which demonstrates that the introduction of FMOF can alleviate the degradation of PEO. In addition, in the N 1*s* (Fig. 5g) and F 1*s* (Fig. 5h) of PEO-FMOF, high content inorganic component of Na_3_N and NaF resulted from decomposition of TFSI^−^ anions was detected, indicating an inorganic salt-rich SEI layer was formed after introducing FMOF. By contrast, in PEO-SPE, the content of CF_3_ is much higher than that of NaF, which indicates a small amount of decomposition of TFSI^−^ anions and difficult to form a stable and robust passivating layer, leading to the continuous decomposition of PEO [44]. The suppressed polymer degradation and prior decomposition of TFSI^−^ in the PEO-FMOF electrolyte are attributed to an anion-rich Na^+^ coordinated structure induced by FMOF.

### Electrochemical Properties of PEO-FMOF in ASSMBs

**Fig. 6:**
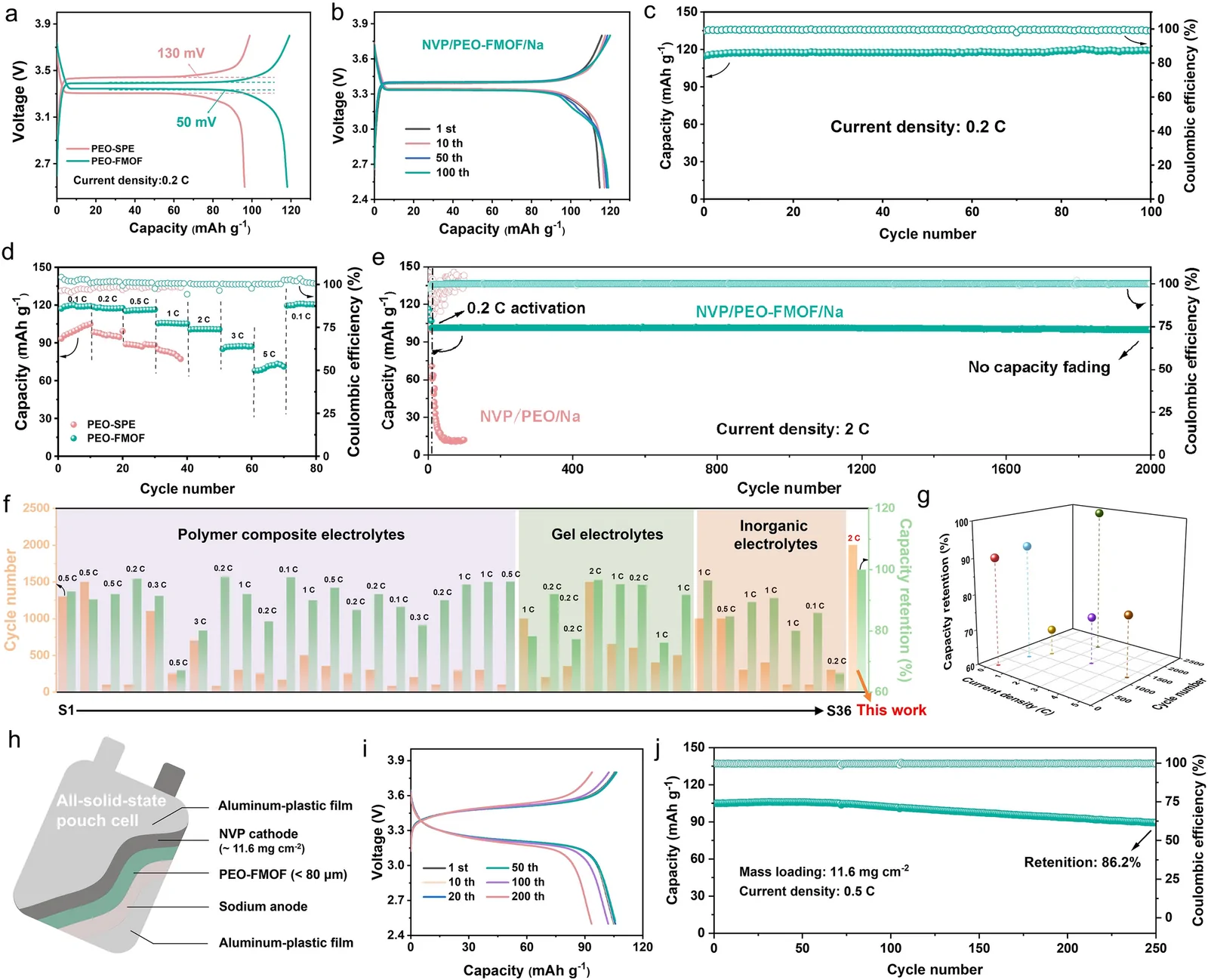
Electrochemical performances of ASSMBs. **a** Charge/discharge profiles of the first cycle of NVP/Na cells under 0.2 C. **b**, **c** Charge/discharge profiles of different cycles and cycling performance of NVP/PEO-FMOF/Na cell under 0.2 C. **d** Rate capability comparison of NVP/Na cells. **e** Long-term cycling performance of NVP/Na cells under 2 C. **f** Comparison of electrochemical properties of currently reported SMBs using different types of electrolytes. **g** Cycle number and capacity retention of NVP/PEO-FMOF/Na at different current densities. **h** Schematic diagram of NVP/Na pouch cell. **i**, **j** Charge/discharge profiles and cycling performance of high-loading NVP/Na pouch cell under 0.5 C

In view of the excellent sodium-ion transport and interfacial stability of PEO-FMOF, we further exploited its feasibility for practical application in ASSMBs involving NVP cathode. Figure S18 displays the cyclic voltammogram (CV) curves at sweep rate of 0.1 mV s^−1^ in the voltage window of 2.7–3.8 V of the full cells using PEO-SPE and PEO-FMOF. The NVP/PEO-FMOF/Na exhibits very small oxidation potential polarization, indicating the superior Na + migration kinetics. Figure 6a shows the initial charge/discharge profiles of the ASSMBs using the PEO-SPE and PEO-FMOF electrolytes at 0.2 C. The PEO-FMOF-based cell exhibits flat potential plateaus with an initial polarization voltage of only 50 mV, much lower than that of the cell using PEO-SPE (130 mV). The polarization voltage of the cell with PEO-FMOF has almost no change with the cycling number increase (Fig. 6b). Due to the rapid ionic migration kinetics and stable electrolyte/sodium metal interface, the all-solid-state NVP/Na cell with PEO-FMOF possesses high discharge capacity and excellent cycling performance (Fig. 6c).

Furthermore, the rate capability of the cells with different electrolytes was evaluated with the current density ranging from 0.1 to 5 C. As indicated in Fig. 6d, the specific discharge capacity of NVP/PEO-FMOF/Na cell is much higher than those of the cell using PEO-SPE electrolyte under the same conditions. Notably, when the current return to 0.1 C, although a high-capacity retention was obtained, the Coulombic efficiency exceeds 100%. This abnormal phenomenon may be essentially a capacity compensation effect caused by the “reparative release” of interface problems accumulated in high current density cycles at low current density. Considering the advantages of the PEO-FMOF in improving the rate properties of the ASSMBs, the cycling performance of the cell using this electrolyte was further evaluated under high current densities. Impressively, the NVP/PEO-FMOF/Na cell displays an outstanding cycling performance, delivering a long lifespan for over 2000 cycles with nearly 100% high-capacity retention even at elevated current rate of 2 C, while the capacity of NVP/PEO/Na cell is extremely attenuated in the initial stage (Fig. 6e). Such cycling performance metric surpasses most of currently literature-reported results using various solid-state electrolytes under comparable conditions (Fig. 6f and Table S2). In addition, we also tested its cycling performance under different current densities (Figs. S19–S23), as expected, excellent cycling performance was achieved in the PEO-FMOF-based all-solid-state full cells, and the results are summarized in Fig. 6g. The impressive cycling performance and rate capability should be attributed to the facilitated Na^+^ migration ability and highly stable SEI layer in NVP/PEO-FMOF/Na cell.

To further verify the promising applicability of the PEO-FMOF, the NVP/Na pouch cell with high cathode mass loading was assembled using a facile hot-pressing method (Fig. 6h). Figures 6i, j and S24 provide the cycling performance of pouch cells using high-loading integrated cathode at different current densities of 0.2 and 0.5 C. Even under a high NVP mass loading up to 11.6 mg cm^−2^, the cell using PEO-FMOF electrolyte still displayed excellent cycling stability with a capacity retention of 86.2% after 250 cycles at 0.5 C. In addition, as shown in Fig. S25, the excellent overall performance allowed us to fabricate a foldable pouch cell that can continuously light up light-emitting diodes (LEDs) under folding and cutting operations, suggesting that the PEO-FMOF prepared in this work possesses high safety at the practically pouch cell level.

## Conclusions

In summary, a fluorinated MOF was introduced into PEO-based SPEs to modulate sodium salt dissociation behavior, coordination structure, and interfacial chemistry through electrostatic engineering. The FMOF with abundant F atoms acted as a powerful electric-rich centrosome, shows electrostatic attractive interaction for Na^+^, promoting the dissociation of sodium salts to form more free Na^+^. Meanwhile, due to the electrostatic repulsion interactions with TFSI^−^, the FMOF forces the anions to participate in the solvation structure to form an anion-rich weak Na–O coordination structure for facilitating the Na^+^ transport and regulating the interfacial chemistry. As a result, the PEO-FMOF electrolytes show a high ionic conductivity (1.01 mS cm^−1^), a high Na^+^ transference number (0.78), and excellent interfacial stability. Benefiting from these advantages, the symmetric cell with this PEO-FMOF yields stable Na plating/stripping cycling over 2500 h at 0.1 mA cm^−2^. The assembled ASSMBs deliver excellent cycling stability, showing a long life over 2000 cycles even at 2 C with capacity retention nearly ~ 100%. Additionally, the pouch cell assembled with a high cathode loading of 11.6 mg cm^−2^, also showed outstanding electrochemical performances. This work provides vital insight into the electrostatic engineering in effective modulation of Na^+^ coordination environment of SPEs for developing high-performance ASSMBs.

## Supplementary Information

Below is the link to the electronic supplementary material.

Supplementary file1 (DOCX 9342 KB)
