# Supplementary material for: Electrostatic Regulation of Na+ Coordination Chemistry for High-Performance All-Solid-State Sodium Batteries
Source: Nanomicro Lett. 2025 Sep 22;18:72. doi: 10.1007/s40820-025-01910-1 (PMC12450857; doi:10.1007/s40820-025-01910-1)
Supplement: Supplementary file 1 — Supplementary file1 (DOCX 9342 KB) [file 40820_2025_1910_MOESM1_ESM.docx]

Supporting Information for

**Electrostatic Regulation of Na^+^ Coordination Chemistry for High-Performance All-Solid-State Sodium Batteries**

Penghui Song^1^, Suli Chen^1^*, Junhong Guo^1^, Junchen Wu^1^, Qiongqiong Lu^2^*, Haijiao Xie^3^, Qingsong Wang^4^*, Tianxi Liu^1^

*^1^* The Key Laboratory of Synthetic and Biological Colloids, Ministry of Education, School of Chemical and Material Engineering, Jiangnan University, Wuxi 214122, P. R. China

*^2^* Institute of Materials, Henan Key Laboratory of Advanced Conductor Materials, Henan Academy of Sciences, Zhengzhou 450046, P. R. China

*^3^* Hangzhou Yanqu Information Technology Co., Ltd., Hangzhou, 310003, P. R. China

*^4^* Bavarian Center for Battery Technology (BayBatt), Department of Chemistry, University of Bayreuth, Universitätsstr. 30, 95447 Bayreuth, Germany

***Corresponding authors. E-mail: [luqq@hnas.ac.cn](mailto:luqq@hnas.ac.cn) (Qiongqiong Lu); [qingsong.wang@uni-bayreuth.de](mailto:qingsong.wang@uni-bayreuth.de) (Qingsong Wang); [chensl@jiangnan.edu.cn](mailto:chensl@jiangnan.edu.cn) (Suli Chen)

**S1 Calculation Method**

Density Functional Theory (DFT) Calculations: The binding energy (E_b_) were performed with Gaussian16, A03 software package. The geometry optimization calculations were performed using the Lee-Yang-Parr correlation functional (B3LYP) and def2-SVP basis including Grimme dispersion corrections (GD3BJ). Then, the singlet point energy calculations were based on the B3LYP functional and a larger def2-TZVP basis including GD3BJ. The binding energy (E_b_) was calculated by the following equation:

$$E_{b}=E_{\mathrm{Co}m\mathrm{plex}}-\left( E_{M1}+E_{M2} \right)$$

where $E_{\mathrm{Co}m\mathrm{plex}}$, $E_{M1}$, and $E_{M2}$ represent the free energies of the complex, and free energies of the interacting molecules, respectively.

The electrostatic potential (ESP) figures were calculated using the CP2K code with a mixed Gaussian and planewave basis sets [S1]. Core electrons were represented with norm-conserving Goedecker-Teter-Hutter pseudopotentials [S2-S4], and the valence electron wavefunction was expanded in a double-zeta basis set with polarization functions [S5] along with an auxiliary plane wave basis set with an energy cutoff of 450 Ry. The generalized gradient approximation exchange-correlation functional of Perdew, Burke, and Enzerhof (PBE) [S6] was used. Each configuration was optimized with the Broyden-Fletcher-Goldfarb-Shanno (BGFS) algorithm with SCF convergence criteria of 1.0 × 10^-6^ au.

Molecular dynamics (MD) simulation was performed through Gromacs2018.8 software [S7] to study the solvated structure. The force field parameters of PEO, Na^+^ and TFSI^-^ ion were obtained with OPLS-AA force field, the degree of polymerization of PEO is 8. The force field parameters of UIO-66 were obtained with UFF4MOF force field. Atomic charges of all ions were multiplied by scale factor 0.8 to correct the polarization effect of ions. The simulation boxes were generated by packmol software [S8]. These simulation boxes were constructed as follows: 1) System 1 consist of 2 UIO-66, 80 NaTFSI and 160 PEO molecules. 2) System 2 consists of 80 NaTFSI and 160 PEO molecules.

All simulation systems were first energetically minimized using the steepest descent algorithm. Atomistic simulations were further performed in a canonical ensemble (NVT) for 10 ns, and simulation trajectories were recorded at an interval of 100 fs for further structural and dynamical analysis. The VMD software [S9] was used to visualize the systems and obtain the ion association state. The self-diffusion coefficient D was estimated using the last part of the MSD profile based on the equation:

*D* ***=*** $\frac{1}{N}\sum_{i=1}^{N} \frac{\Delta MSD(t)}{6\Delta t}$

Where t (time interval) is the time and the slope of MSD represents the diffusion coefficient.

**S2 Supplementary Table and Figures**

**
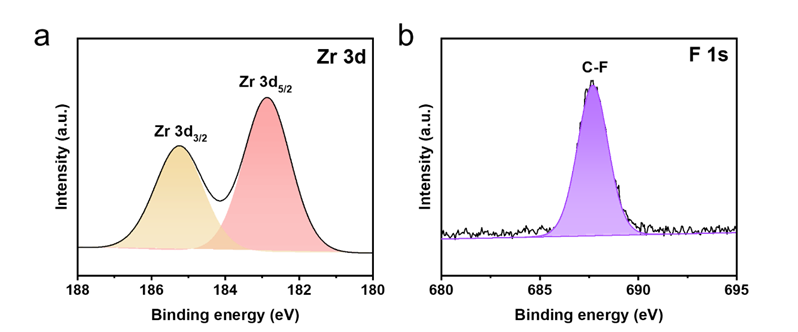
**

**Fig. S1** XPS spectra of UiO-66-(F)_4_: **a** Zr 3d and **b** F 1s


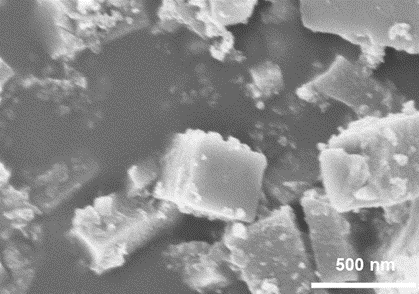


**Fig. S****2** SEM image of UiO-66-(F)_4_


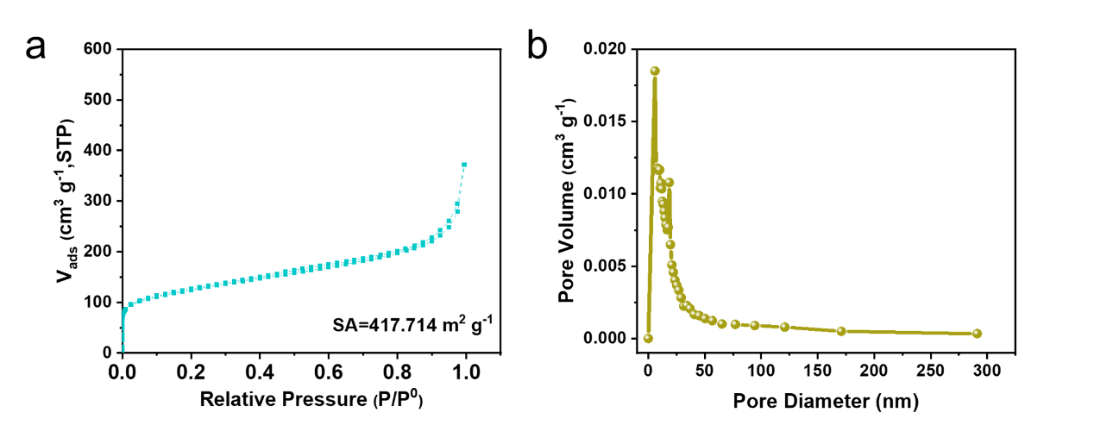


**Fig. S3** N_2_ adsorption-desorption isotherms of UiO-66-(F)_4_


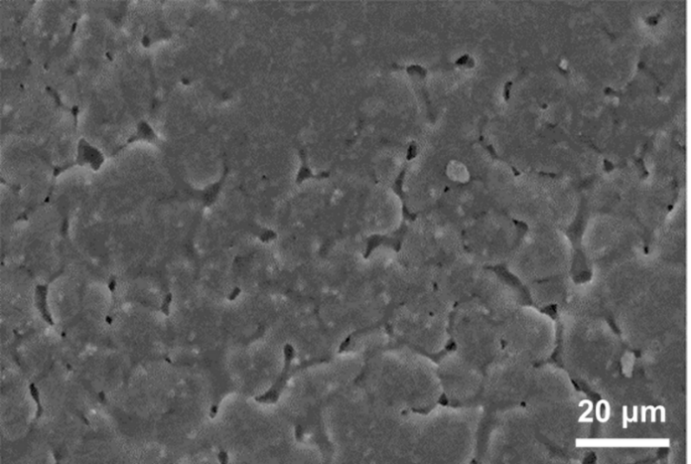


**Fig. S4** SEM image of the obtained PEO-SPE membrane


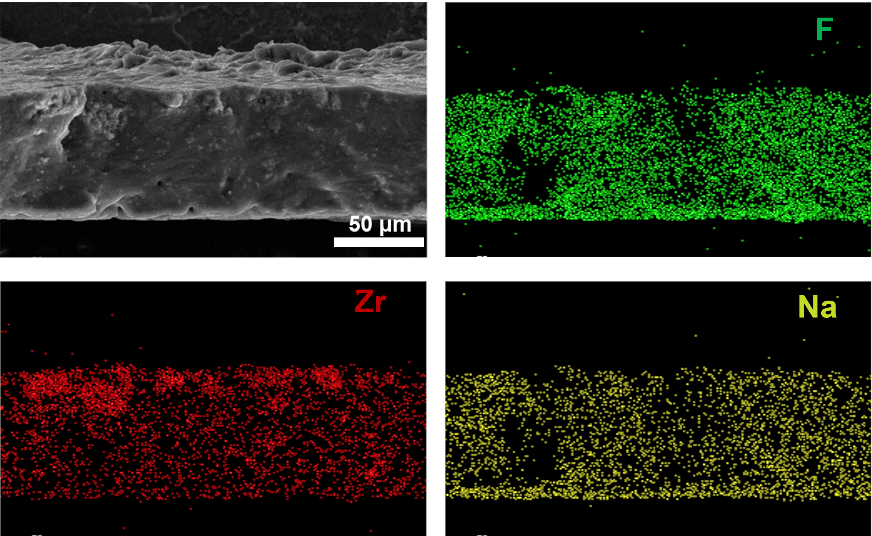


**Fig. S5** Corresponding EDS-mapping (F, Zr and Na elements) images


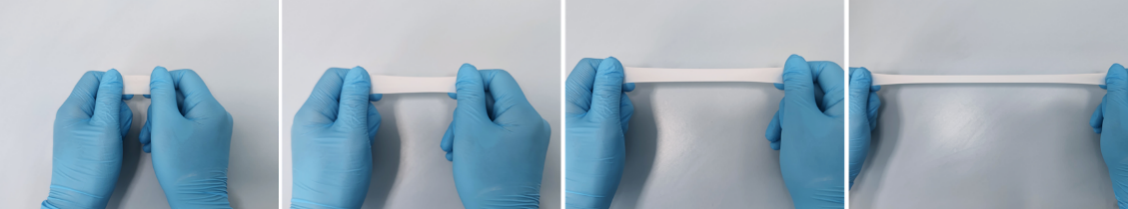


**Fig. S6** Mechanical flexibility display of the PEO-FMOF


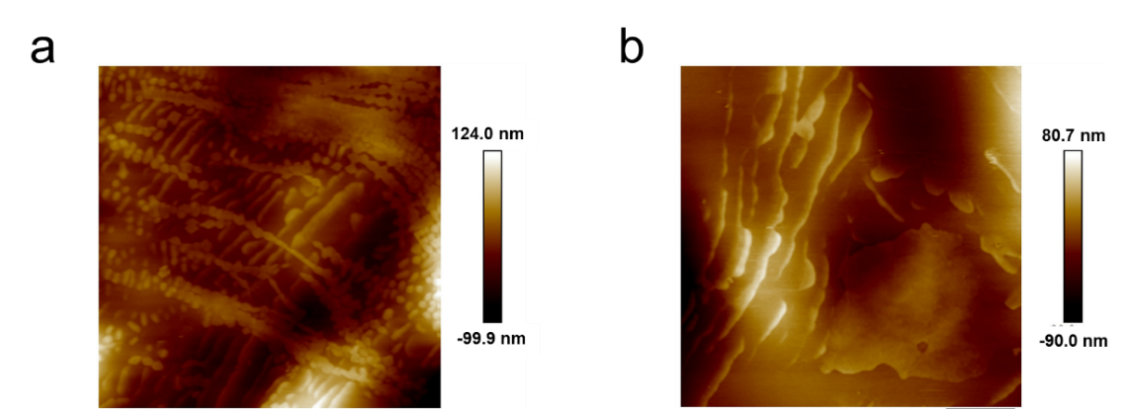


**Fig. S7** **a, b** Morphology from AFM test of PEO-SPE and PEO-FMOF membranes

**Table S1** The melting enthalpy and crystallinity of PEO-SPE and PEO-FMOF with different mass concentrations

| Samples | **Melting enthalpy(J/g)** | **Crystallinity (%)** |
| --- | --- | --- |
| PEO-SPE | 44.86 | 30.0 |
| PEO-FMOF-6 | 38.22 | 27.1 |
| PEO-FMOF-9 | 33.84 | 24.7 |
| PEO-FMOF-12 | 35.93 | 26.9 |


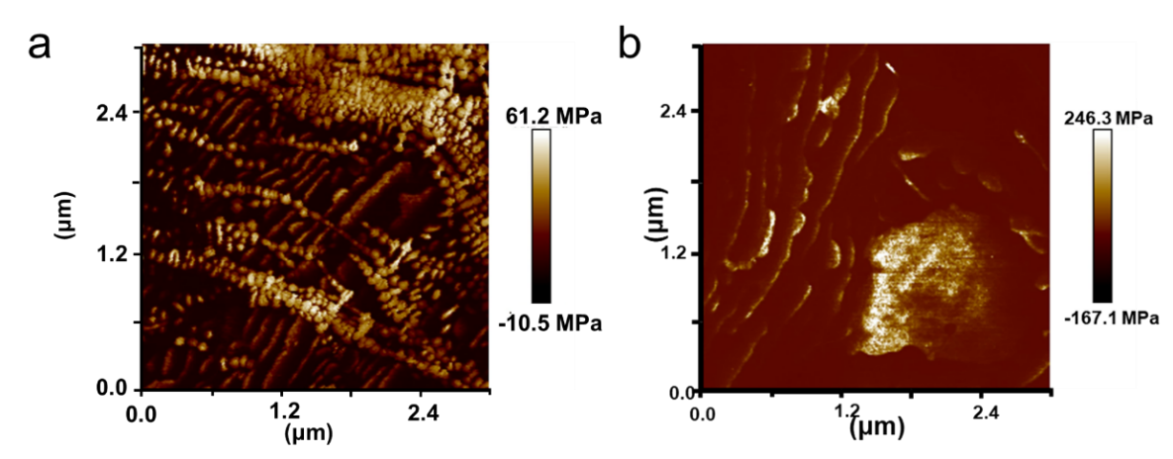


**Fig. S8** **a, b** Young’s modulus results from AFM test of PEO-SPE and PEO-FMOF membranes


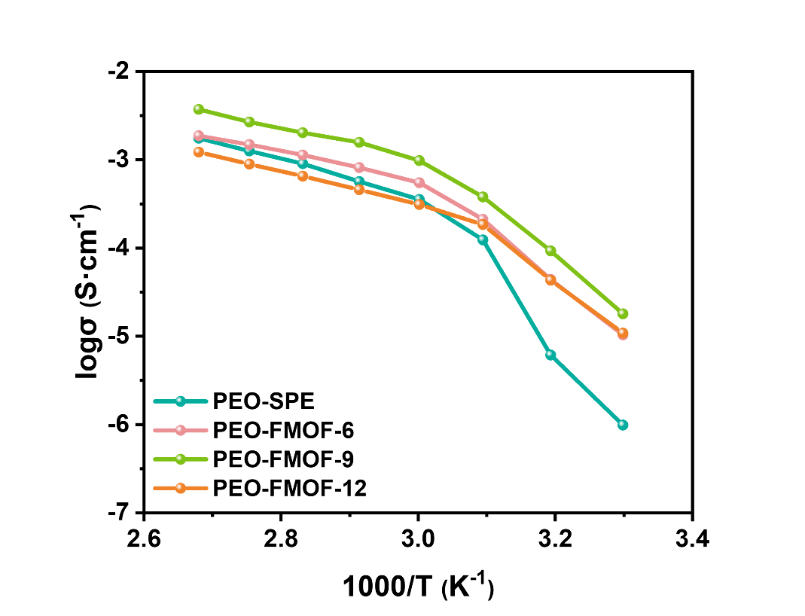


**Fig. S9** Temperature dependency comparison of ionic conductivity for different electrolytes from 30 to 100 °C


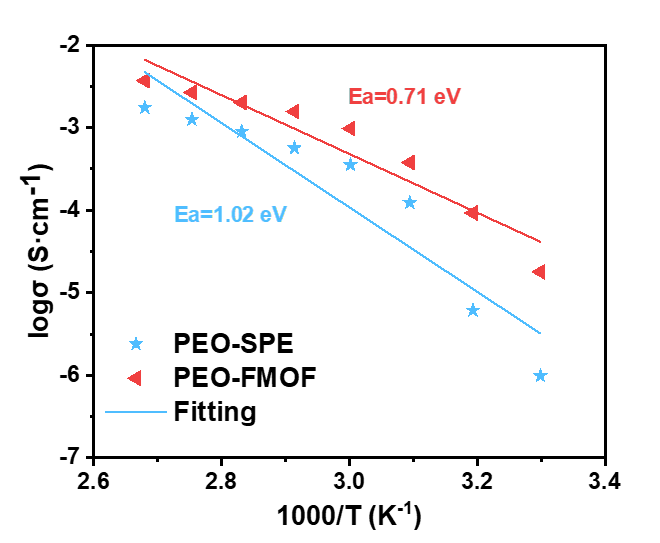


**Fig. S10** Ion migration activation energy of the PEO-SPE and PEO-FMOF


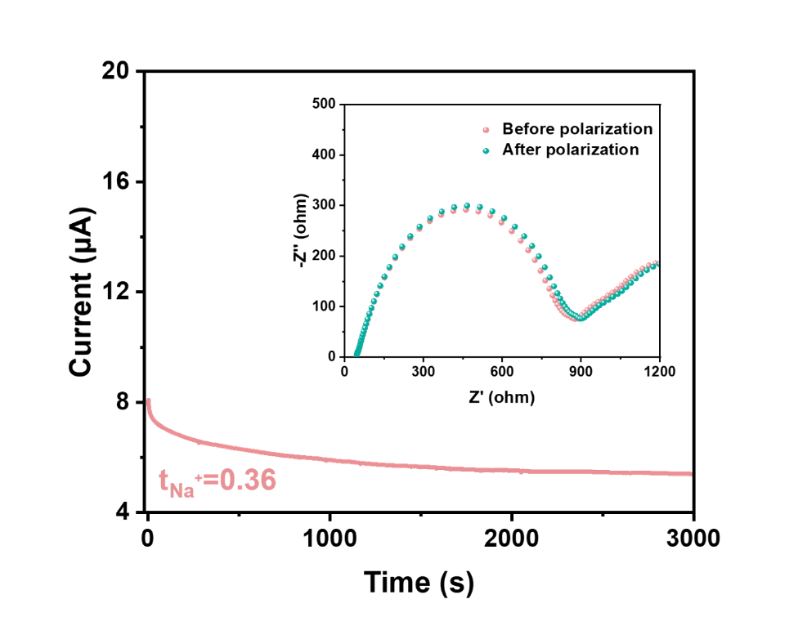


**Fig. S11** Polarization curve of PEO-SPE (The inset is fitted EIS before and after polarization of PEO-SPE).

**
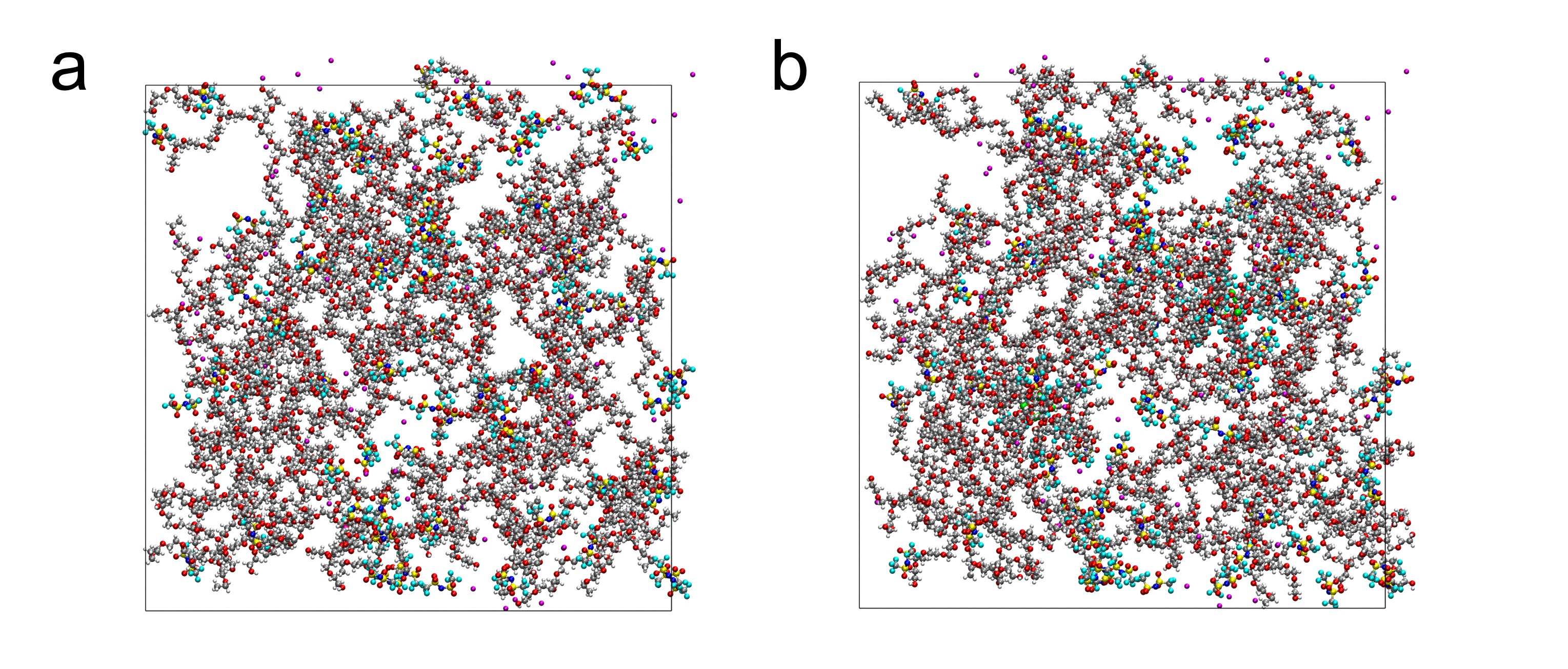
**

**Fig. S12** 3D snapshot of (a) PEO-SPE and (b) PEO-FMOF obtained from MD simulations


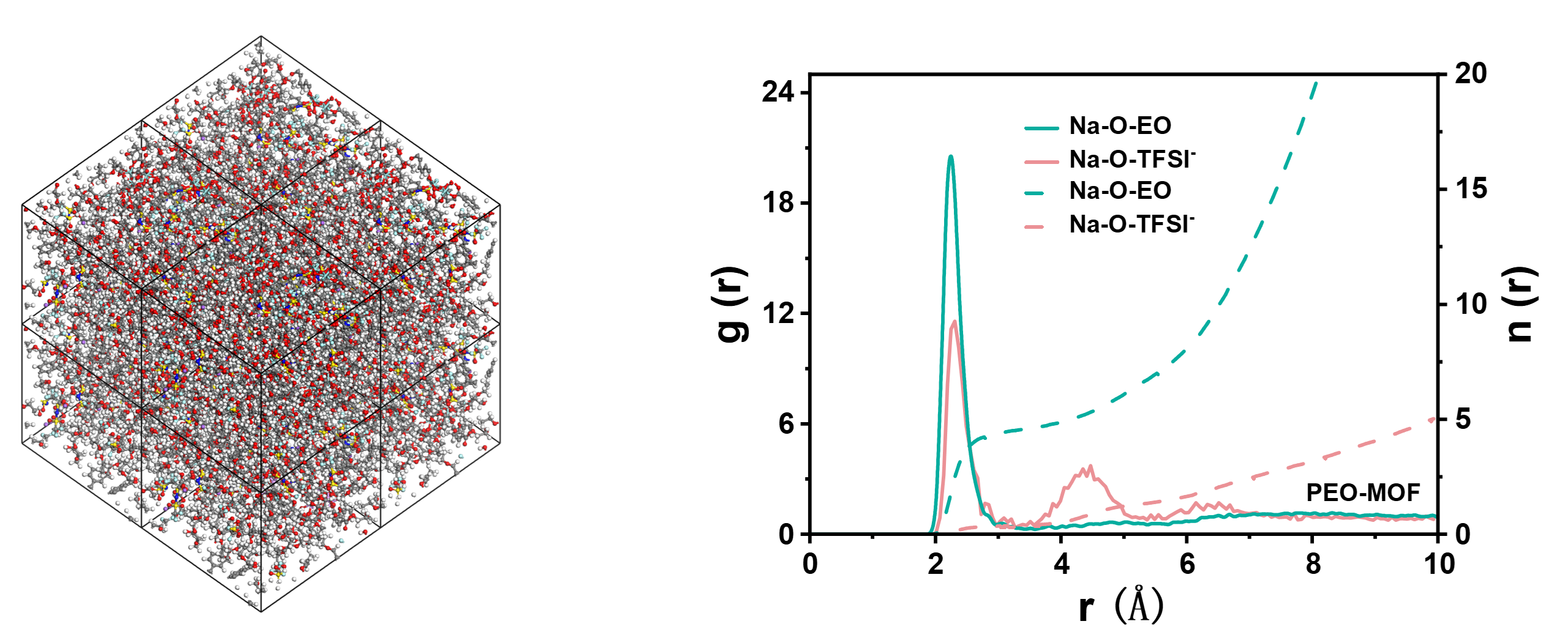


**Fig. S13** 3D snapshot and the corrsponding radial distribution functions of PEO-MOF obtained from MD simulations


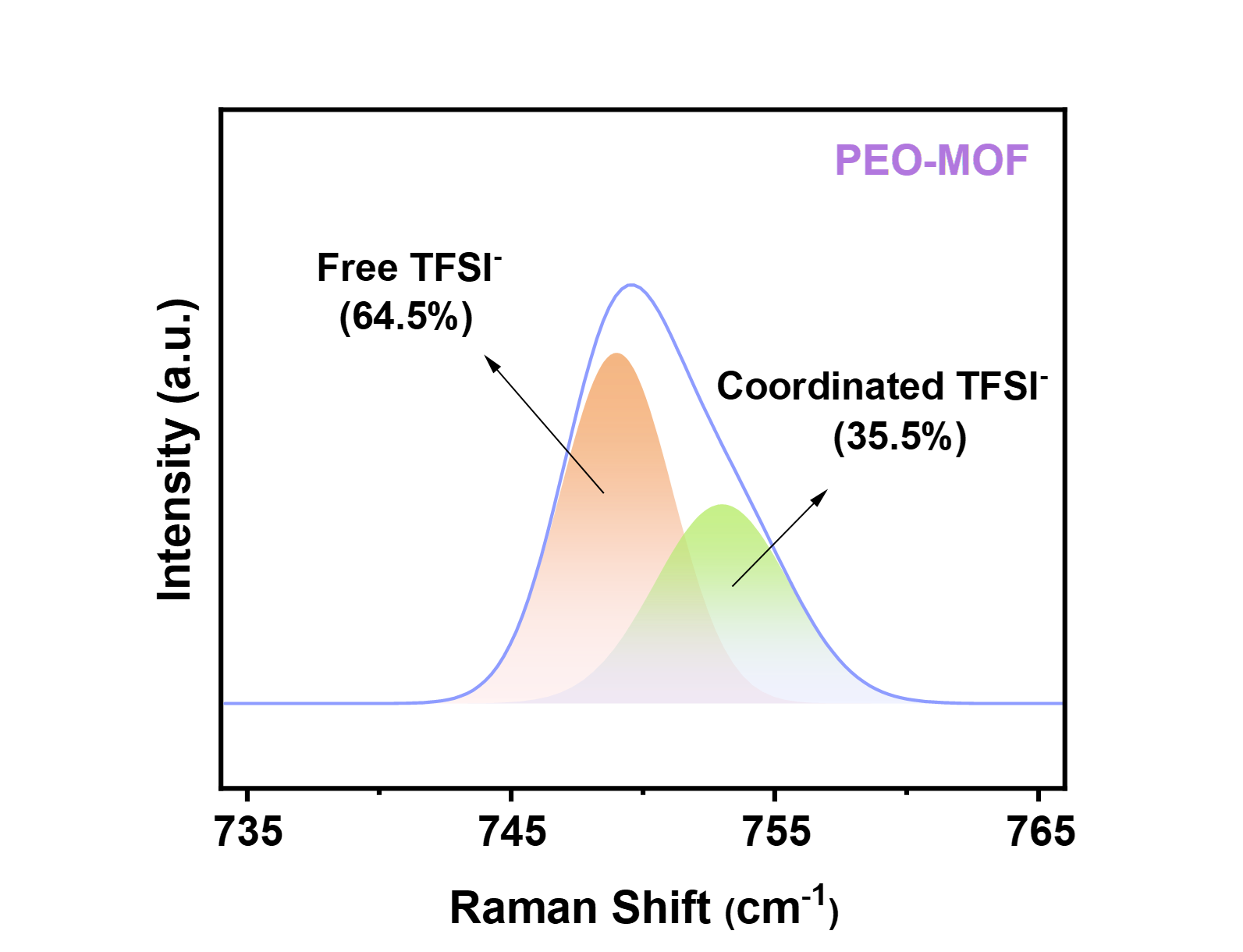


**Fig. S14** Raman spectra of PEO-MOF electrolyte


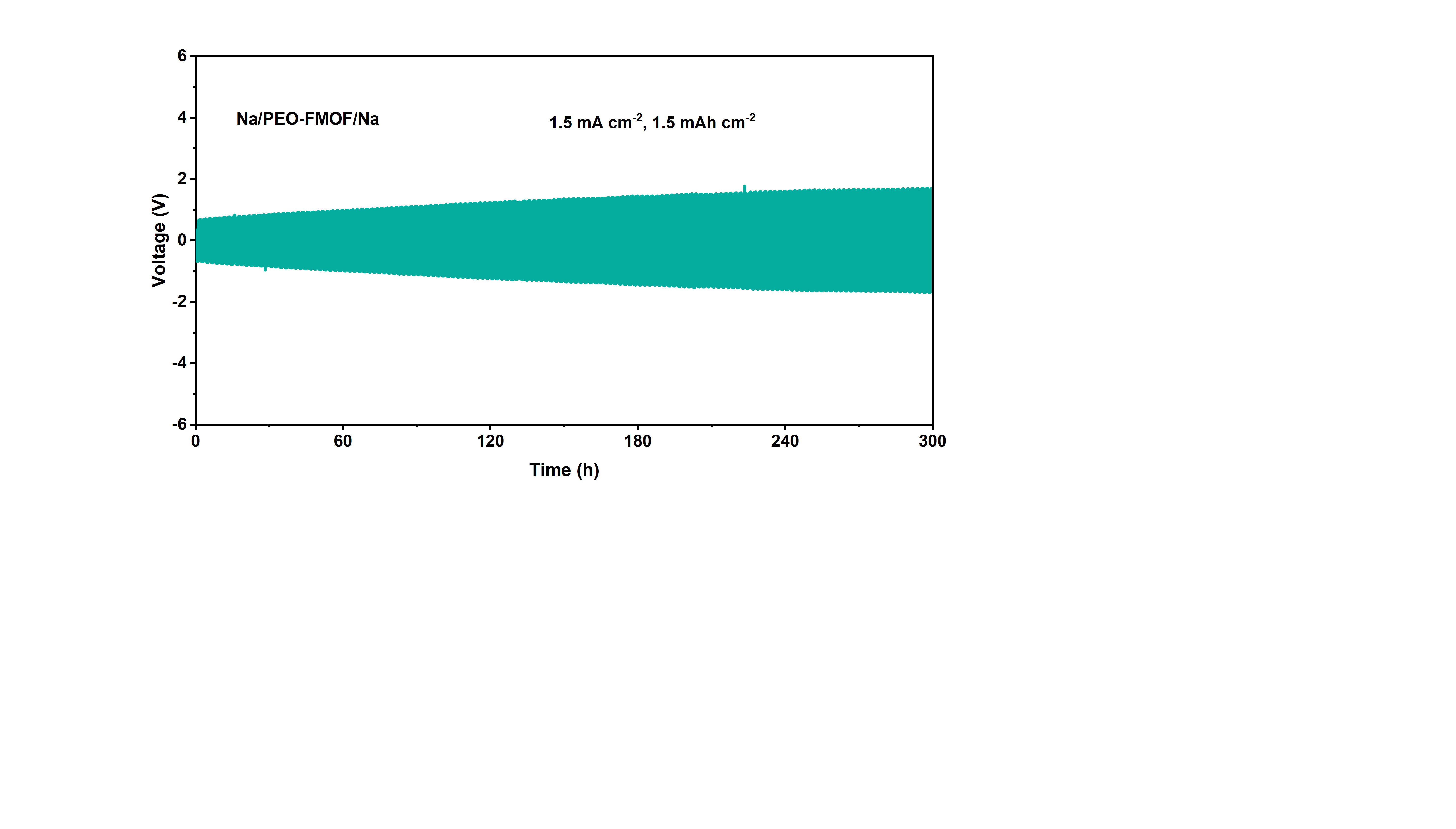


**Fig. S15** Long-term cycling performance of Na/Na symmetric cells using PEO-FMOF at 1.5 mA cm^−2^ and 1.5 mA h cm^−2^


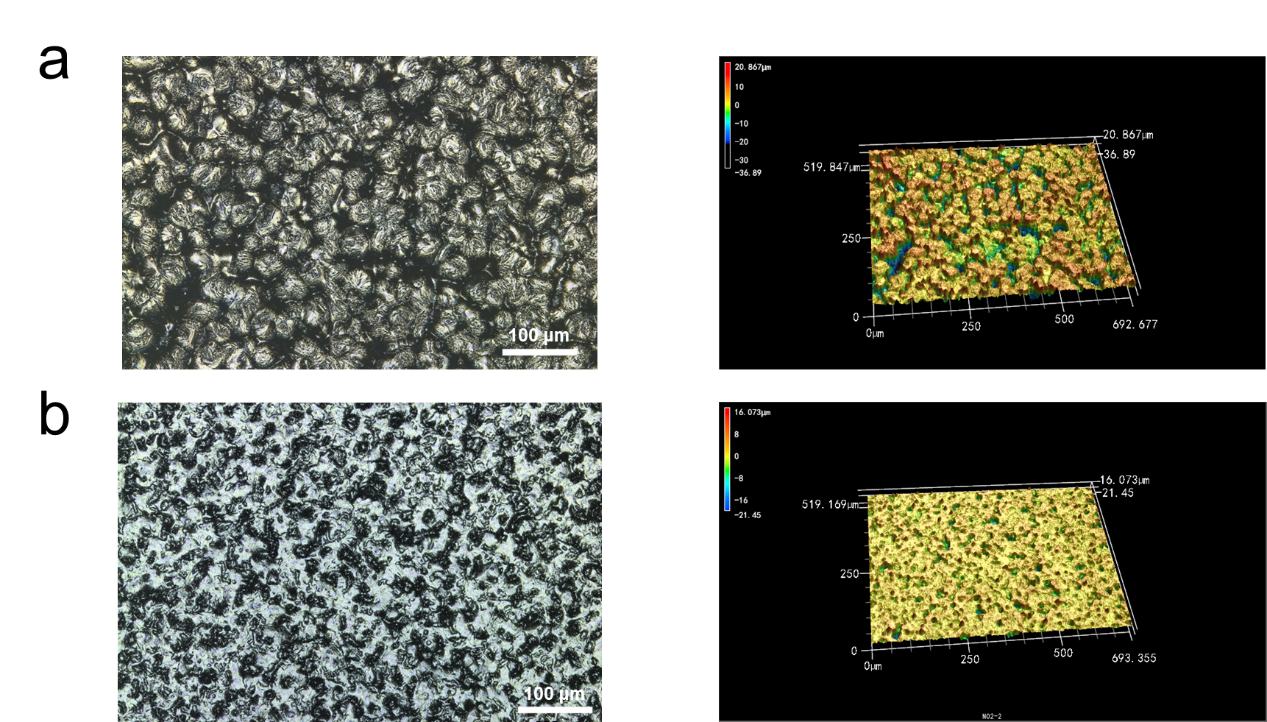


**Fig. S16** Surface profile analysis and 3D morphology reconstruction of cycled electrolyte membrane with **a** PEO-SPE and **b** PEO-FMOF via 3D CLSM


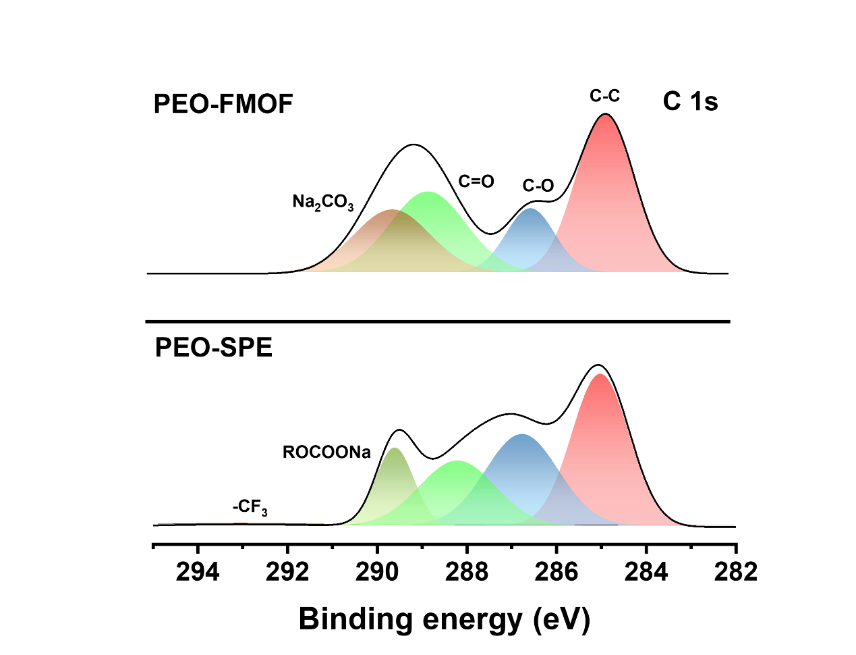


**Fig. S17** XPS spectra of Na foils after cycling with the PEO-SPE and PEO-FMOF: C 1s


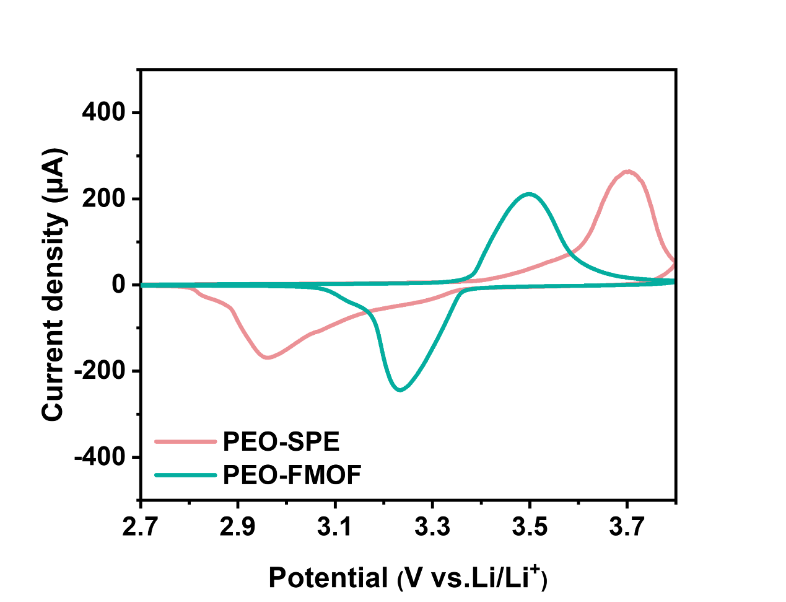


**Fig. S18** CV curves of NVP/Na cells with PEO-SPE and PEO-FMOF

**Table S2** Comparison of different types of electrolytes-based solid-state SMBs

| **Cell** | **Solid-state electrolytes** | **Temperature** | **Current density** | **Cycle number** | **Capacity retention** | **Refs.** |
| --- | --- | --- | --- | --- | --- | --- |
| NVP/Na | PEO-FMOF | 60 ℃ | 2 C | 2000 | 100% | **This work** |
| **Composite electrolytes** | | | | | | |
| NaMNTO/Na | G-NZC_0.05_SPF_0.7_-G | 25℃ | 0.5 C | 1300 | 92.8% | [S10] |
| NVP/Na | PAAEM-SnF@IA | 26℃ | 0.5 C | 1500 | 90.2% | [S11] |
| Te-C/Na | NSS-CMC | RT | 0.5 C | 100 | 88~92% | [S12] |
| NaFePO_4_/Na | PEO-SPC-NZSP | 60 ℃ | 0.2 C | 100 | 97 % | [S13] |
| NVP/Na | GFPP | 50 ℃ | 0.3 C | 1100 | 91.4 % | [S14] |
| NVP/Na | PVDF-SiO_2_ | 60 ℃ | 0.5 C | 250 | 67 % | [S15] |
| c-NFM/Na | B-CPE | 40 ℃ | 3 C | 700 | 80.1 % | [S16] |
| NVP/Na | NASICON-PEO | 80 ℃ | 0.2 C | 80 | 97.6 % | [S17] |
| NVP/Na | DSPE-20 | 23 ℃ | 1 C | 300 | 92 % | [S18] |
| NVP/Na | PE-PEO | 60 ℃ | 0.2 C | 250 | 83 % | [S19] |
| NVP/Na | KNN-NZSP | RT | 0.1 C | 165 | 97.4 % | [S20] |
| NVP/Na | PEO-P-N | 60 ℃ | 1 C | 500 | 90 % | [S21] |
| NVP/Na | α-Al_2_O_3_-PMA/PEG | 70 ℃ | 0.5 C | 350 | 94.1 % | [S22] |
| c-NFN/Na | PVC-CPE | 25 ℃ | 0.2 C | 250 | 86.8 % | [S23] |
| NVP/Na | PMH9-1/1-3NaTFSI | RT | 0.2 C | 300 | 92 % | [S24] |
| NaNFM/Na | CD-HSPE3 | 60 ℃ | 0.1 C | 80 | 87.8 % | [S25] |
| HC/Na | PVDF-co-HFP/SiO_2_ | 25 ℃ | 0.3 C | 200 | 81.8 % | [S26] |
| NVP/Na | NTO CPE | 25 ℃ | 0.2 C | 100 | 90 % | [S27] |
| NVP/Na | PVDF-25wt% NZSP | RT | 1 C | 290 | 95.1 % | [S28] |
| NVP/Na | PEGMEM-co-SSS@ZIF-8 | 80 ℃ | 1 C | 300 | 96 % | [S29] |
| NLM/Na | 3D-15PNZSPP | 30 ℃ | 0.5 C | 100 | 96.1 % | [S30] |
| NVP/Na | ATFPE | 60 ℃ | 1 C | 1000 | 78.2 % | [S31] |
| NNM/Na | CPE-CQDs | 25 ℃ | 0.2 C | 200 | 92 % | [S32] |
| FeHCF/Na | PEPA@NC | 60 ℃ | 0.2 C | 350 | 77.2 % | [S33] |
| **Gel electrolytes** | | | | | | |
| NVP/Na | AS-NFCGE | 25℃ | 2 C | 1500 | 96.6% | [S34] |
| NVP/Na | PPE-50 | 25℃ | 1 C | 650 | 95.2% | [S35] |
| NVP/Na | PLA-NaF | 25℃ | 0.2 C | 600 | 95.0% | [S36] |
| NMNO/Na | SDL-QSPE | RT | 1 C | 400 | 76.1% | [S37] |
| NVP/Na | AT-FCGE | RT | 1 C | 500 | 91.7% | [S38] |
| NVP/Na | AFGPE | RT | 1 C | 1000 | 96.4 % | [S39] |
| NVP/Na | HFP-PC-FEC-Sn | 30 ℃ | 0.5 C | 1000 | 84.7% | [S40] |
| **Inorganic electrolytes** | | | | | | |
| NVP/Na | Na_3.4_Zr_1.6_Sc_0.4_Si_2_PO_12_ | 30℃ | 1 C | 300 | 89.4% | [S41] |
| Me-NVMP/Na | NZSP | 25℃ | 0.5C~1C | 400 | 90.7% | [S42] |
| NCO/Na_3_Sn | NSWS-NZCO | 25℃ | 1 C | 100 | 80% | [S43] |
| Na_3_V_2_(PO_4_)_2_O_2_F/Na | Na_4_B_36_H_34_-7Na_2_B_12_H_12_ | 25℃ | 0.1 C | 100 | 85.8% | [S44] |
| NVP/Na | NASICON | RT | 0.2 C | 300 | 66% | [S45] |


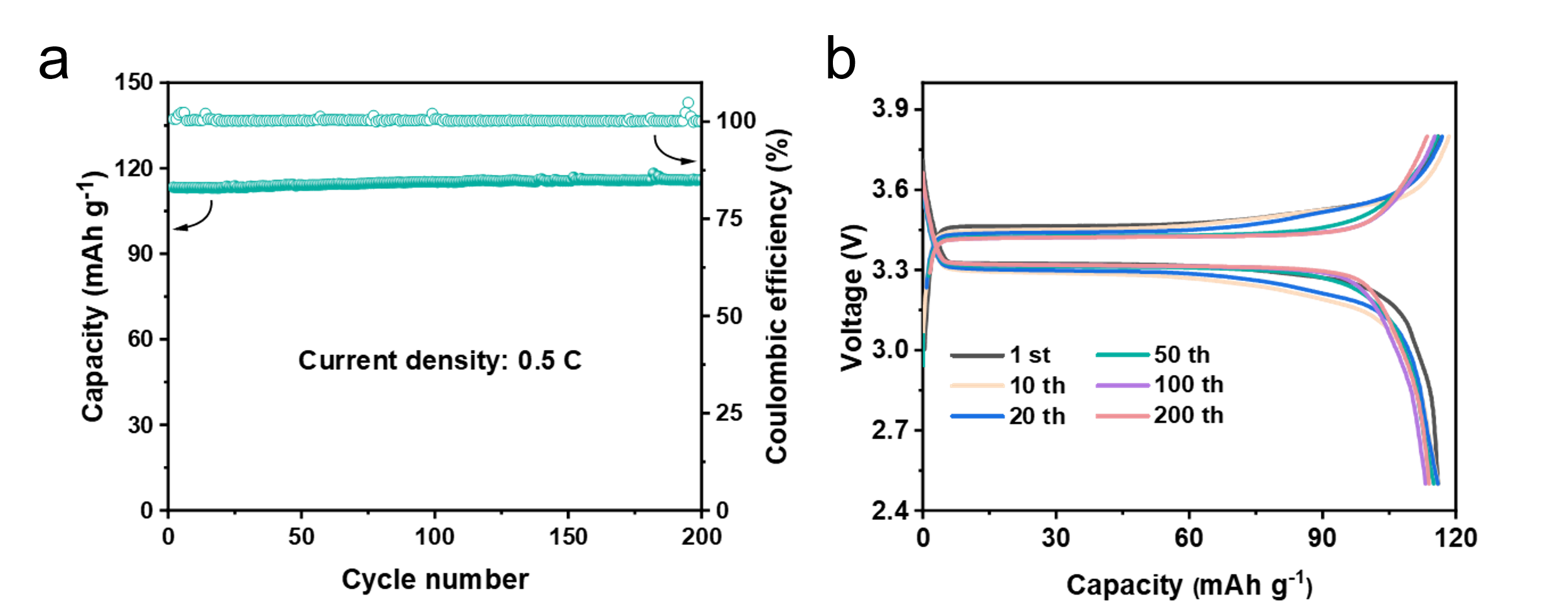


**Fig. S19 a** Cycling performance and **b** charge/discharge profiles of different cycles of NVP/PEO-FMOF/Na cell at 0.5 C at 60 ℃


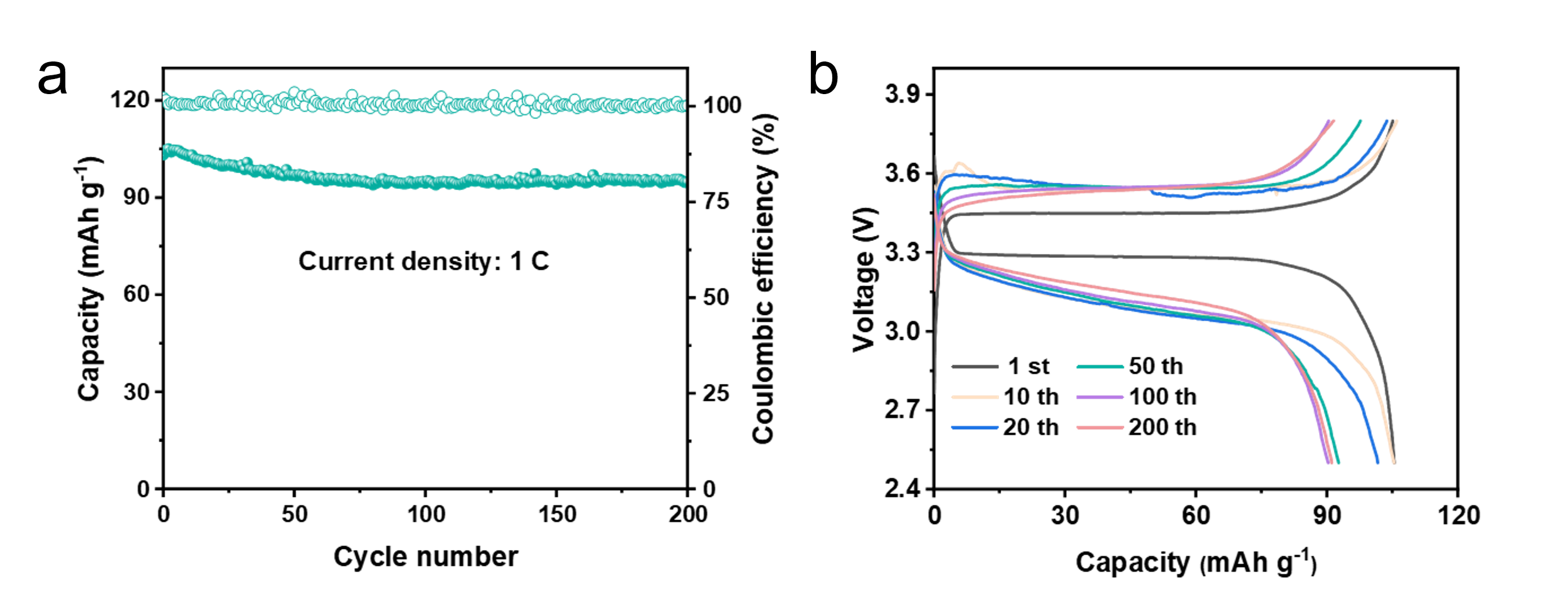


**Fig. S20 a** Cycling performance and **b** charge/discharge profiles of different cycles of NVP/PEO-FMOF/Na cell at 1 C at 60 ℃


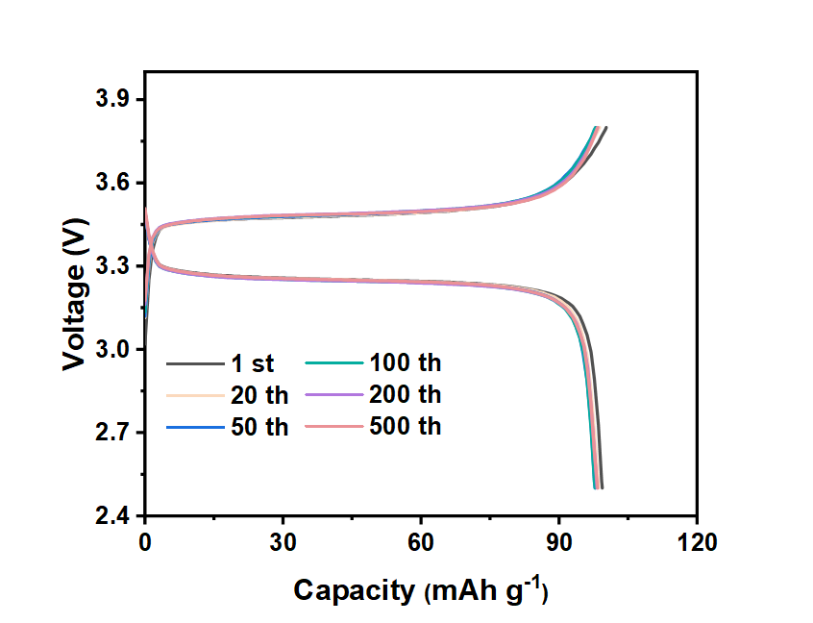


**Fig. S21** Charge/discharge profiles of different cycles of NVP/PEO-FMOF/Na cell at 2 C at 60 ℃


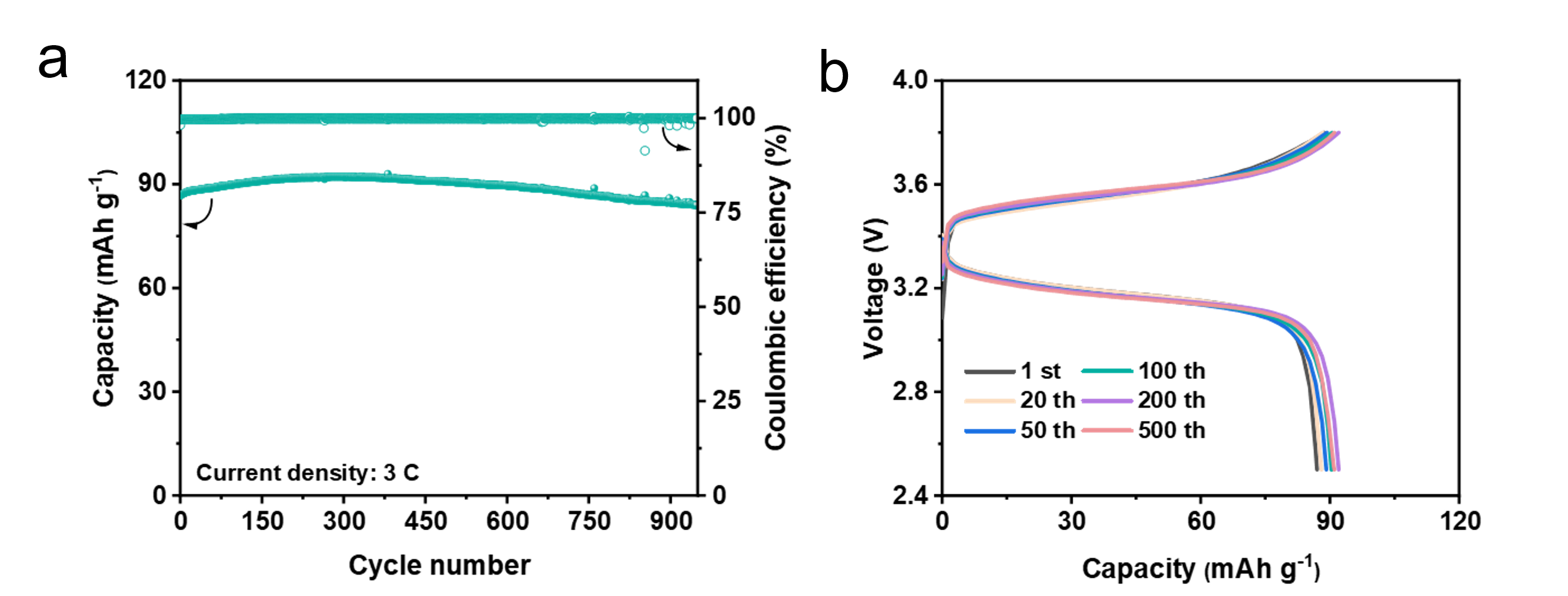


**Fig. S22** **a** Cycling performance and **b** charge/discharge profiles of different cycles of NVP/PEO-FMOF/Na cell at 3 C at 60 ℃


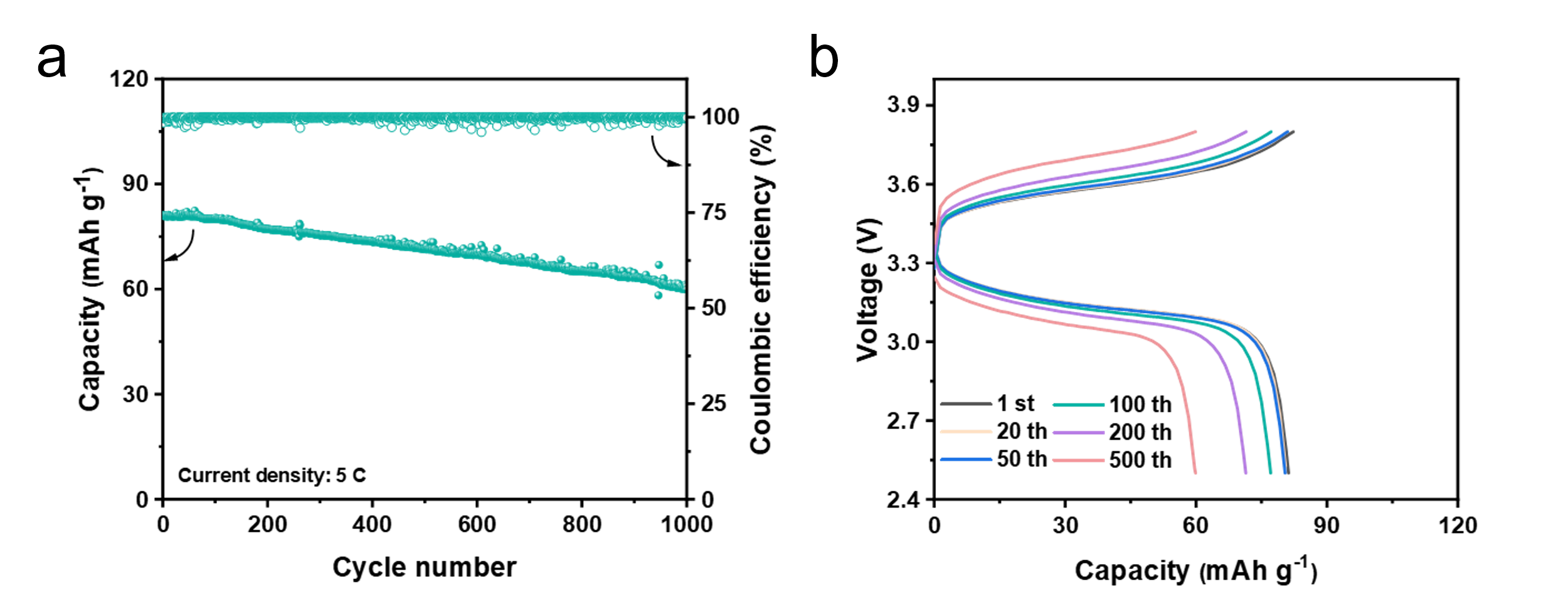


**Fig. S23** **a** Cycling performance and **b** charge/discharge profiles of different cycles of NVP/PEO-FMOF/Na cell at 5 C at 60 ℃


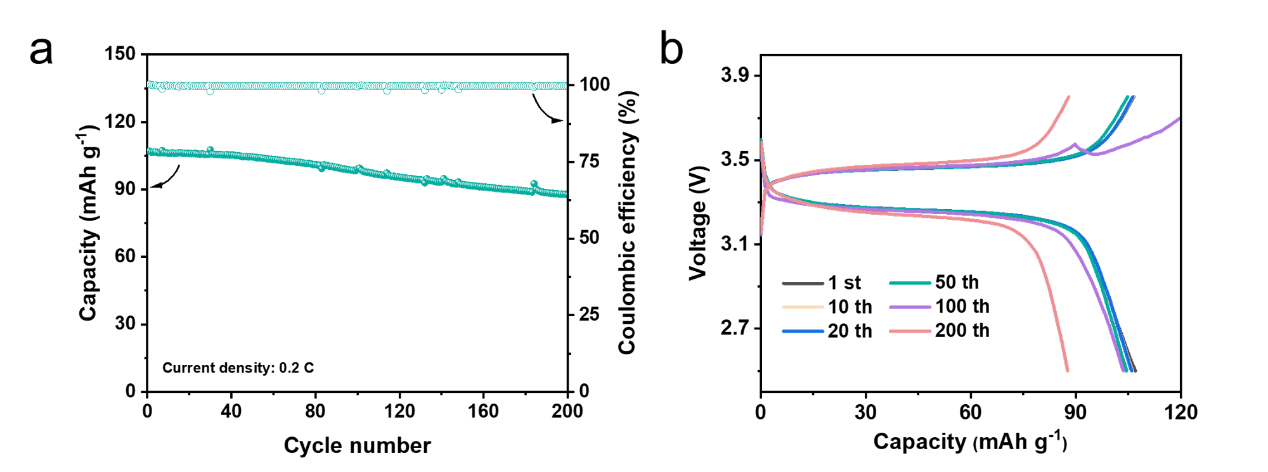


**Fig. S24** **a** Cycling performance and **b** charge/discharge profiles of different cycles of high-loading NVP/PEO-FMOF/Na pouch cell at 0.2 C at 60 ℃


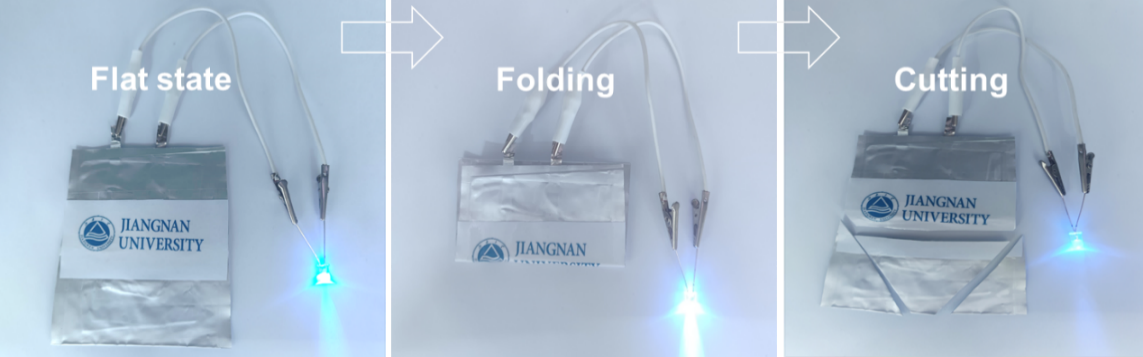


**Fig. S25** The assembled NVP/PEO-FMOF/Na pouch cell lights up the LED board at the flat, folding, and cutting states

**Supplementary References**

1. J. VandeVondele, M. Krack, F. Mohamed, M. Parrinello, T. Chassaing et al., Quickstep: Fast and accurate density functional calculations using a mixed Gaussian and plane waves approach. Comput. Phys. Commun. **167**(2), 103–128 (2005). <https://doi.org/10.1016/j.cpc.2004.12.014>
2. S. Goedecker, M. Teter, J. Hutter, Separable dual-space Gaussian pseudopotentials. Phys. Rev. B **54**(3), 1703–1710 (1996). <https://doi.org/10.1103/physrevb.54.1703>
3. C. Hartwigsen, S. Goedecker, J. Hutter, Relativistic separable dual-space Gaussian pseudopotentials from H to Rn. Phys. Rev. B **58**(7), 3641–3662 (1998). <https://doi.org/10.1103/physrevb.58.3641>
4. M. Krack, M. Parrinello, All-electron ab-initio molecular dynamics. Phys. Chem. Chem. Phys. **2**(10), 2105–2112 (2000). <https://doi.org/10.1039/b001167n>
5. J. VandeVondele, J. Hutter, Gaussian basis sets for accurate calculations on molecular systems in gas and condensed phases. J. Chem. Phys. **127**(11), 114105 (2007). <https://doi.org/10.1063/1.2770708>
6. J. Perdew, K. Burke, M. Ernzerhof, Generalized gradient approximation made simple. Phys. Rev. Lett. **77**(18), 3865–3868 (1996). <https://doi.org/10.1103/PhysRevLett.77.3865>
7. M.J. Abraham, T. Murtola, R. Schulz, S. Páll, J.C. Smith et al., GROMACS: High performance molecular simulations through multi-level parallelism from laptops to supercomputers. SoftwareX **1–2**, 19–25 (2015). <https://doi.org/10.1016/j.softx.2015.06.001>
8. L. Martínez, R. Andrade, E.G. Birgin, J.M. Martínez, PACKMOL: a package for building initial configurations for molecular dynamics simulations. J. Comput. Chem. **30**(13), 2157–2164 (2009). <https://doi.org/10.1002/jcc.21224>
9. W. Humphrey, A. Dalke, K. Schulten, VMD: Visual molecular dynamics. J. Mol. Graph. **14**(1), 33–38 (1996). <https://doi.org/10.1016/0263-7855(96)00018-5>
10. W. Wang, W. Yuan, Z. Zhao, P. Zhou, P. Zhang et al., Sandwiched composite electrolyte with excellent interfacial contact for high-performance solid-state sodium-ion batteries. J. Colloid Interface Sci. **652**, 132–141 (2023). <https://doi.org/10.1016/j.jcis.2023.08.052>
11. S. Wu, C. Ji, F. Tang, L. Zhang, K. Fang et al., Nanofiber matrix composite electrolyte for regulating ion distribution in fast kinetic sodium-ion batteries operating at wide temperatures. Energy Storage Mater. **67**, 103308 (2024). <https://doi.org/10.1016/j.ensm.2024.103308>
12. S. Dong, G. Xie, S. Xu, X. Tan, M. Chaudhary et al., Cellulose-encapsulated composite electrolyte design: toward chemically and mechanically enhanced solid-sodium batteries. ACS Nano **18**(25), 16285–16296 (2024). <https://doi.org/10.1021/acsnano.4c03910>
13. M. Kannadasan, K. Sathiasivan, I. Pandurangan, M. Balakrishnan, Synergistic nanocomposite polymer electrolytes for advanced all-solid-state sodium-ion batteries. Int. J. Hydrog. Energy **78**, 634–641 (2024). <https://doi.org/10.1016/j.ijhydene.2024.06.305>
14. C. Luo, Q. Li, D. Shen, R. Zheng, D. Huang et al., Enhanced interfacial kinetics and fast Na^+^ conduction of hybrid solid polymer electrolytes for all-solid-state batteries. Energy Storage Mater. **43**, 463–470 (2021). <https://doi.org/10.1016/j.ensm.2021.09.031>
15. S. Bag, C. Zhou, S. Reid, S. Butler, V. Thangadurai, Electrochemical studies on symmetric solid-state Na-ion full cell using Na_3_V_2_(PO_4_)_3_ electrodes and polymer composite electrolyte. J. Power Sources **454**, 227954 (2020). <https://doi.org/10.1016/j.jpowsour.2020.227954>
16. S. Chen, F. Feng, H. Che, Y. Yin, Z.-F. Ma, High performance solid-state sodium batteries enabled by boron contained 3D composite polymer electrolyte. Chem. Eng. J. **406**, 126736 (2021). <https://doi.org/10.1016/j.cej.2020.126736>
17. Z. Zhang, K. Xu, X. Rong, Y.-S. Hu, H. Li et al., Na_3.4_Zr_1.8_Mg_0.2_Si_2_PO_12_ filled poly(ethylene oxide)/Na(CF_3_SO_2_)_2_N as flexible composite polymer electrolyte for solid-state sodium batteries. J. Power Sources **372**, 270–275 (2017). <https://doi.org/10.1016/j.jpowsour.2017.10.083>
18. H.M. Law, J. Yu, S.C.T. Kwok, G. Zhou, M.J. Robson et al., A hybrid dual-salt polymer electrolyte for sodium metal batteries with stable room temperature cycling performance. Energy Storage Mater. **46**, 182–191 (2022). <https://doi.org/10.1016/j.ensm.2022.01.001>
19. J. Zhang, Y. Su, Y. Qiu, X. Zhang, F. Xu et al., High-strength, thin, and lightweight solid polymer electrolyte for superior all-solid-state sodium metal batteries. ACS Appl. Mater. Interfaces **16**(23), 30128–30136 (2024). <https://doi.org/10.1021/acsami.4c05023>
20. Y. Wang, Z. Wang, F. Zheng, J. Sun, J.A.S. Oh et al., Ferroelectric engineered electrode-composite polymer electrolyte interfaces for all-solid-state sodium metal battery. Adv. Sci. **9**(13), 2105849 (2022). <https://doi.org/10.1002/advs.202105849>
21. E. Matios, H. Wang, J. Luo, Y. Zhang, C. Wang et al., Reactivity-guided formulation of composite solid polymer electrolytes for superior sodium metal batteries. J. Mater. Chem. A **9**(34), 18632–18643 (2021). <https://doi.org/10.1039/D1TA05490B>
22. X. Zhang, X. Wang, S. Liu, Z. Tao, J. Chen, A novel PMA/PEG-based composite polymer electrolyte for all-solid-state sodium ion batteries. Nano Res. **11**(12), 6244–6251 (2018). <https://doi.org/10.1007/s12274-018-2144-3>
23. S. Chen, H. Che, F. Feng, J. Liao, H. Wang et al., Poly(vinylene carbonate)-based composite polymer electrolyte with enhanced interfacial stability to realize high-performance room-temperature solid-state sodium batteries. ACS Appl. Mater. Interfaces **11**(46), 43056–43065 (2019). <https://doi.org/10.1021/acsami.9b11259>
24. G. Chen, L. Ye, K. Zhang, M. Gao, H. Lu et al., Hyperbranched polyether boosting ionic conductivity of polymer electrolytes for all-solid-state sodium ion batteries. Chem. Eng. J. **394**, 124885 (2020). <https://doi.org/10.1016/j.cej.2020.124885>
25. S. Chen, F. Feng, Y. Yin, H. Che, X.-Z. Liao et al., A solid polymer electrolyte based on star-like hyperbranched β-cyclodextrin for all-solid-state sodium batteries. J. Power Sources **399**, 363–371 (2018). <https://doi.org/10.1016/j.jpowsour.2018.07.096>
26. A. Das, A. Melepurakkal, P. Sreeram, K.T. Gireesh, N.T.M. Balakrishnan et al., Exceptional cyclability of thermally stable PVdF-*co*-HFP/SiO_2_ nanocomposite polymer electrolytes for sodium ion batteries. J. Energy Storage **73**, 109026 (2023). <https://doi.org/10.1016/j.est.2023.109026>
27. X. Ma, Y. Liu, Y. Zhang, Y. Gong, Layered sodium titanate with a matched lattice: a single ion conductor in a solid-state sodium metal battery. Chem. Sci. **14**(47), 13812–13824 (2023). <https://doi.org/10.1039/d3sc04355j>
28. Y. Ruan, X. Huang, H. Lei, Y. Liu, J. Wang et al., A flexible and free-standing composite solid electrolyte with a pomegranate-like structure for solid-state sodium-ion batteries. Solid State Ion. **403**, 116406 (2023). <https://doi.org/10.1016/j.ssi.2023.116406>
29. J. Zhang, Y. Wang, Q. Xia, X. Li, B. Liu et al., Confining polymer electrolyte in MOF for safe and high-performance all-solid-state sodium metal batteries. Angew. Chem. Int. Ed. **63**(16), e202318822 (2024). <https://doi.org/10.1002/anie.202318822>
30. W. Wang, M. Ding, S. Chen, J. Weng, P. Zhang et al., A novel composite solid electrolyte with ultrahigh ion transference number and stability for solid-state sodium metal batteries. Chem. Eng. J. **491**, 151989 (2024). <https://doi.org/10.1016/j.cej.2024.151989>
31. J. Guo, F. Feng, S. Zhao, R. Wang, M. Yang et al., Achieving ultra-stable all-solid-state sodium metal batteries with anion-trapping 3D fiber network enhanced polymer electrolyte. Small **19**(16), 2206740 (2023). <https://doi.org/10.1002/smll.202206740>
32. Y. Zhang, H. Zheng, H. Ding, K.A. Jabbar, L. Gao et al., Ceria quantum dot filler-modified polymer electrolytes for three-dimensional-printed sodium solid-state batteries. Polymers **16**(12), 1707 (2024). <https://doi.org/10.3390/polym16121707>
33. T. Wang, M. Zhang, K. Zhou, H. Wang, A. Shao et al., A hetero-layered, mechanically reinforced, ultra-lightweight composite polymer electrolyte for wide-temperature-range, solid-state sodium batteries. Adv. Funct. Mater. **33**(22), 2215117 (2023). <https://doi.org/10.1002/adfm.202215117>
34. M. Yang, F. Feng, J. Guo, R. Wang, J. Yu et al., Anion trapping-coupling strategy driven asymmetric nonflammable gel electrolyte for high performance sodium batteries. Energy Storage Mater. **70**, 103492 (2024). <https://doi.org/10.1016/j.ensm.2024.103492>
35. Y. Li, B. Wei, J. Yu, D. Chen, Multiple Na+ transport pathways and interfacial compatibility enable high-capacity, room-temperature quasi-solid sodium batteries. J. Colloid Interface Sci. **666**, 447–456 (2024). <https://doi.org/10.1016/j.jcis.2024.04.047>
36. X. Guo, Z. Xie, R. Wang, J. Luo, J. Chen et al., Interface-compatible gel-polymer electrolyte enabled by NaF-solubility-regulation toward all-climate solid-state sodium batteries. Angew. Chem. Int. Ed. **63**(18), e202402245 (2024). <https://doi.org/10.1002/anie.202402245>
37. J. Pan, Y. Zhang, F. Sun, M. Osenberg, A. Hilger et al., Designing solvated double-layer polymer electrolytes with molecular interactions mediated stable interfaces for sodium ion batteries. Angew. Chem. Int. Ed. **62**(17), e202219000 (2023). <https://doi.org/10.1002/anie.202219000>
38. M. Yang, F. Feng, Y. Ren, S. Chen, F. Chen et al., Coupling anion-capturer with polymer chains in fireproof gel polymer electrolyte enables dendrite-free sodium metal batteries. Adv. Funct. Mater. **33**(46), 2305383 (2023). <https://doi.org/10.1002/adfm.202305383>
39. Z. Liu, R. Wang, J. Yu, Z. Miao, Z. Xu et al., Asymmetric fireproof gel polymer electrolyte constructed by boron-contained covalent organic framework for dendrite-free sodium metal battery. Nano Res. **17**(11), 9679–9687 (2024). <https://doi.org/10.1007/s12274-024-6910-0>
40. Z. Yang, H. Jiang, X. Li, X. Liang, J. Wei et al., Fabricating wide-temperature-range quasi-solid sodium batteries with fast ion transport *via* tin additives. Adv. Funct. Mater. **34**(45), 2407713 (2024). <https://doi.org/10.1002/adfm.202407713>
41. Q. Wang, C. Yu, L. Li, X. Liu, X. Zhang et al., Sc-doping in Na_3_Zr_2_Si_2_PO12 electrolytes enables preeminent performance of solid-state sodium batteries in a wide temperature range. Energy Storage Mater. **54**, 135–145 (2023). <https://doi.org/10.1016/j.ensm.2022.10.026>
42. Y. Li, C. Sun, Z. Sun, M. Li, H. Jin et al., Boosting Na-O affinity in Na_3_Zr_2_Si_2_PO_12_ electrolyte promises highly rechargeable solid-state sodium batteries. Adv. Funct. Mater. **34**(40), 2403937 (2024). <https://doi.org/10.1002/adfm.202403937>
43. L. Hu, H. Li, F. Chen, Y. Liu, J. Wang et al., Overcoming the Na-ion conductivity bottleneck for the cost-competitive chloride solid electrolytes. J. Energy Chem. **95**, 1–8 (2024). <https://doi.org/10.1016/j.jechem.2024.03.023>
44. M. Jin, D. Xu, Z. Su, Z. He, X. Chen et al., A practical nonflammable Na_4_B_36_H_34_-based hydroborate electrolyte for high-voltage all-solid-state sodium batteries. ACS Energy Lett. **9**(3), 1176–1183 (2024). <https://doi.org/10.1021/acsenergylett.4c00112>
45. P.W. Jaschin, C.R. Tang, E.D. Wachsman, High-rate cycling in 3D dual-doped NASICON architectures toward room-temperature sodium-metal-anode solid-state batteries. Energy Environ. Sci. **17**(2), 727–737 (2024). <https://doi.org/10.1039/D3EE03879C>
